# Scalable inference of cell differentiation networks in gene therapy clonal tracking studies of haematopoiesis

**DOI:** 10.1093/bioinformatics/btad605

**Published:** 2023-09-29

**Authors:** Luca Del Core, Danilo Pellin, Ernst C Wit, Marco A Grzegorczyk

**Affiliations:** University of Groningen – Bernoulli Institute, 9747AG Groningen, The Netherlands; University of Nottingham – School of Mathematical Sciences, Nottingham NG72RD, United Kingdom; Harvard Medical School, Boston, MA 02115, United States; University of Groningen – Bernoulli Institute, 9747AG Groningen, The Netherlands; Università della Svizzera italiana – Institute of Computing, 6962 Lugano, Switzerland; University of Groningen – Bernoulli Institute, 9747AG Groningen, The Netherlands

## Abstract

**Motivation:**

Investigating cell differentiation under a genetic disorder offers the potential for improving current gene therapy strategies. Clonal tracking provides a basis for mathematical modelling of population stem cell dynamics that sustain the blood cell formation, a process known as haematopoiesis. However, many clonal tracking protocols rely on a subset of cell types for the characterization of the stem cell output, and the data generated are subject to measurement errors and noise.

**Results:**

We propose a stochastic framework to infer dynamic models of cell differentiation from clonal tracking data. A state-space formulation combines a stochastic quasi-reaction network, describing cell differentiation, with a Gaussian measurement model accounting for data errors and noise. We developed an inference algorithm based on an extended Kalman filter, a nonlinear optimization, and a Rauch-Tung-Striebel smoother. Simulations show that our proposed method outperforms the state-of-the-art and scales to complex structures of cell differentiations in terms of nodes size and network depth. The application of our method to five *in vivo* gene therapy studies reveals different dynamics of cell differentiation. Our tool can provide statistical support to biologists and clinicians to better understand cell differentiation and haematopoietic reconstitution after a gene therapy treatment. The equations of the state-space model can be modified to infer other dynamics besides cell differentiation.

**Availability and implementation:**

The stochastic framework is implemented in the R package Karen which is available for download at https://cran.r-project.org/package=Karen. The code that supports the findings of this study is openly available at https://github.com/delcore-luca/CellDifferentiationNetworks.

## 1 Introduction

Haematopoiesis is the process responsible for maintaining the number of circulating blood cells that are undergoing continuous turnover. This process has a tree-like structure with haematopoietic stem cells (HSCs) at the root node (Cooper and Adams 2023). Each cell division gives rise to progeny cells that can retain the properties of their parent cell (self-renewal) or differentiate, thereby “moving down” the haematopoietic tree. As the progeny cells move further away from the HSCs, their pluripotent ability is increasingly restricted. Clarifying how HSCs differentiate is essential for understanding how they attain specific functions and offers the potential for therapeutic manipulation (Kawamoto *et al.* 2010). Several mathematical models have been proposed to describe haematopoiesis *in vivo*. One of the first stochastic models of haematopoiesis was introduced in the early 1960s suggesting that it is the population as a whole that is regulated rather than individual cells that behave stochastically (Till *et al.* 1964).

Since then, many other works aimed at describing the dynamics of haematopoiesis as a stochastic process. For example, in 2002 a single cell-based stochastic model was proposed to describe the evolution of HSCs as a result of switching between a nonproliferating and a proliferating environment, with transition probabilities, leading to a microenvironment-dependent competition of cells in a stochastic sense (Roeder and Loeffler 2002). By simulating several growth scenarios, the authors were able to describe stem cell kinetics, individual clone tracking, fluctuating clonal contribution, and to compare the theoretical results with experimental findings (Roeder *et al.* 2005). More recently, various studies analysed data generated by advanced lineage tracing protocols to calibrate novel mathematical models of HSCs differentiation. For example, in 2001 an inference method of a two-compartment hidden Markov model of haematopoiesis was proposed (Catlin *et al.* 2001). The model parameters were calibrated on time-series of cellular binary markers from a hybrid cats study. Since data from one of the two compartments were not observed, the parameters were estimated by solving the Kolmogorov forward equations and using a nonlinear least squares inference approach. Later in 2010 a stochastic model of haematopoiesis was proposed to keep track of HSCs differentiation in multiple cell compartments (Dingli and Pacheco 2010): the cells move within a single-branch structure with unknown probability from the smallest bone marrow compartment, housing active HSCs, up to the largest compartment hosting cells that are leaving the bone marrow to enter the circulation. The parameters of this model were calibrated on granulopoiesis data. Subsequently, in 2019 a multidimensional continuous-time Markov model was developed to describe the rates of cell differentiation in a hierarchical fashion (Pellin *et al.* 2019). The likelihood of this model is derived from a local linear approximation of the biochemical Master equation, and the parameters are estimated via a penalized least squares method from clonal tracking data. In late 2019, another stochastic process of haematopoiesis was formulated as a continuous-time, multi-type branching processes, whose parameters were estimated using moment-based techniques from cellular barcoding data (Xu *et al.* 2019). More recently in 2020, a Bayesian networks framework has been proposed to describe cell differentiation (Di Serio *et al.* 2020): the process of haematopoiesis was described through different levels of cell differentiation and the corresponding parameters were estimated using clonal tracking data from gene therapy clinical trials. Some of these methods are able to take into account missing cell types, such as those that are difficult to collect from the bone marrow (Catlin *et al.* 2001, Xu *et al.* 2019), but none considers the bias provided by false-negative clonal tracking errors. The state-of-the-art methods assume that missing clone observations correspond to minimal clones and set the corresponding counts to zero. But this hypothesis is too restrictive, because it does not take into account other technical sources of false-negative errors, such as low-informative sample replicates and threshold detection failure (Kim *et al.* 2019). Besides, it has also been shown that false-negative errors strongly depend on calling pipeline parameters, as well as read coverage (Bobo *et al.* 2016).

To overcome the limitations of the existent approaches, we propose a novel stochastic framework aimed at investigating mechanistic models of cell differentiation from clonal tracking data while cautiously treating all the undetected values as nonmeasurable states. More precisely, we model cell differentiation using a continuous-discrete state-space formulation including a system of Itô-type stochastic differential equations (SDE) describing clonal dynamics, coupled with a measurement model that links the sparse and noisy corrupted measurements to the underlying process’ states. In Section 2, we provide a formal definition of our modelling approach along with an expectation–maximization (E–M) algorithm, based on extended Kalman filtering (EKF) and Rauch–Tung–Striebel (RTS) smoothing, to infer the unknown parameters. In Section 3, we extensively test the method on several simulation studies including a direct comparison with the prior art and we apply our framework to five *in vivo* high dimensional clonal tracking datasets, comparing four biologically plausible models of cell differentiation. In Section 4, we discuss our results from both a methodological and biological perspective.

## 2 Materials and methods

**Figure 1.**
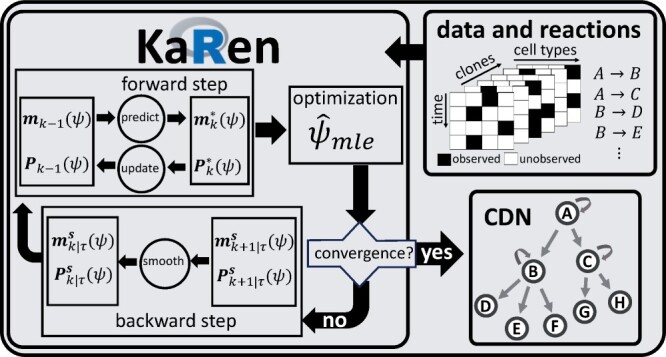
Analysis’ flowchart: a clonal tracking dataset and the biochemical reactions (top-right) are the input of our framework Karen (left). It mainly consists of three parts: a filtering step, a maximum likelihood step, and a smoothing step. These steps are iterated until convergence is reached. The inferred cell differentiation network is returned (bottom-right).

A concise graphical representation of our proposed framework of Kalman reaction networks (Karen) is shown in Fig. 1. The input consists of a clonal tracking dataset, including clone-specific information on the number of cells generated for each lineage over time, and a set of biochemical reactions coding for cell duplication, cell death, and cell differentiation. Inference is done via an E–M algorithm. The E-step is based on a Kalman filter/smoother, aimed at estimating the state variables given the parameters inferred from the M-step. While in the M-step, a nonlinear optimization method updates the unknown parameters given the states estimated by the E-step. Both steps are iterated until convergence is reached. The inferred cell differentiation network is returned as the main output. More details on the input syntax can be found in the documentation of our R package Karen, which we published along with this article. The following subsections provide details on the state-space formulation of cell differentiation and the E–M algorithm.

### 2.1 A stochastic model for cell differentiation

Here, we assume that cell duplication, cell death and cell differentiation can take place from time *t* to time t+Δt in a combinatorial number of ways directly proportional to the cell counts at time *t* and the corresponding rate parameters. This hypothesis is equivalent to the chemical law of mass action (Érdi and Tóth 1989) whereby cell duplication, cell death, and cell differentiation are biochemical reactions that can be properly described by stochastic quasi-reaction networks. Consistently with the definition of a chemical reaction network of Supplementary Section S1, we consider a Markov process


(1) $$x_{t}=(x_{1t},\ldots ,x_{nt}),$$

of one clone and *n* cell types evolving in a time interval (t,t+Δt) according to a set of *K* distinct biochemical reactions whose net-effect vectors ${\left\{v_{k}\right\}}_{k=1}^{K}$ and hazard functions ${\left\{h_{k}(x_{t},\theta )\right\}}_{k=1}^{K}$ are defined as


(2) $$v_{k}=\{\begin{array}{c} (\cdots 1\cdots )\prime \\ i(k)\\ (\cdots -1\cdots )\prime \\ i(k)\\ (\cdots -1\cdots 2\cdots )\prime \\ i(k)j(k) \end{array}h_{k}(x_{t},\theta )=\{\begin{array}{c} x_{i(k)t}\alpha_{i(k)}\\ x_{i(k)t}\delta_{i(k)}\\ x_{i(k)t}\lambda_{i(k)j(k)} \end{array},$$

where *i*(*k*) and *j*(*k*) are the cell types possibly involved in the *k*th reaction, and j(k)∈O(i(k)), where


(3) $$O(i)=\{j|\lambda_{ij}>0\}$$

is called the offspring set of cell type *i*. The hazard functions include a linear growth term $x_{i(k)t}\alpha_{i(k)}$ with duplication rate $\alpha_{i(k)}>0$, a linear term $x_{i(k)t}\delta_{i(k)}$ for cell death with a death rate $\delta_{i(k)}>0$, and a linear term $x_{i(k)t}\lambda_{i(k)j(k)}$ for cell differentiation from cell lineage *i*(*k*) to cell lineage *j*(*k*) with rate $\lambda_{i(k)j(k)}>0$. The vector parameter


(4) $$\theta =\left(\alpha_{1}\cdots \alpha_{n},\delta_{1}\cdots \delta_{n},\lambda \prime_{1O(1)}\cdots \lambda \prime_{nO(n)}\right)\prime ,$$

appearing in the hazard functions, includes all the dynamic parameters, where $\lambda \prime_{iO(i)}$ is the vector of all the differentiation rates from cell lineage *i* to its offspring set O(i). Finally, we define the net-effect matrix and the hazard vector as


(5) $$\begin{array}{c} V=\left[\begin{array}{c} v_{1}\cdots v_{K} \end{array}\right]\in Z^{n\times K},\\ h(x_{t},\theta )=\left(h_{1}(x_{t},\theta ),\ldots ,h_{K}(x_{t},\theta )\right)\prime , \end{array}$$

and, as probabilistic assumption, we use the Kolmogorov-forward ODEs


(6) $$\begin{array}{c} \frac{\partial P(x,t)}{\partial t}=-\nabla_{x}\left\{\mu (x;\theta )P(x;t)\right\}+\frac{1}{2}\nabla_{x}^{2}\left\{\beta (x;\theta )P(x;t)\right\},\\ \mu (x;\theta )=\mathrm{Vh}(x;\theta ),\beta (x;\theta )=V\left[\begin{array}{ccc} h_{1}(x_{t};\theta ) & & \\ & \ddots & \\ & & h_{K}(x_{t};\theta ) \end{array}\right]V\prime , \end{array}$$

obtained from a continuous approximation of the Master equation (see details in Supplementary Section S2).

### 2.2 State-space formulation

Since the aim of this work is to calibrate the parameters of the continuous-time stochastic model defined by Equations (1–6) on clonal tracking data that have been collected at discrete time points, we use a continuous-discrete state-space model (Del Core 2023). In this formulation the dynamic component is the system of Itô-type SDEs


(7) $$\begin{array}{c} dx=\mu (x;\theta )dt+\beta {\left(x;\theta \right)}^{1/2}dW,\\ dW∼N_{n}(0,dtI_{n}), \end{array}$$

where μ(x;θ) and β(x;θ) are defined by Equations (1–6), combined with the measurement model


(8) $$\begin{array}{c} y_{t}=G_{t}x_{t}+r_{t},\;r_{t}∼N_{d}(0,R_{t}),\\ R_{t}=\rho_{0}I_{d}+\rho_{1}\left[\begin{array}{ccc} {\left(G_{k}x_{t}\right)}_{1} & & \\ & \ddots & \\ & & {\left(G_{k}x_{t}\right)}_{d} \end{array}\right],\;d\leq n, \end{array}$$

where $G_{t}$ is a *d *×* n* matrix selecting only the measurable states of $x_{t}$ subject to an additive noise $r_{t}$, and $x_{t}$ is a shorthand notation for x(t). The covariance matrix $R_{t}$ models the measurement noise as a linear function $G_{t}x_{t}$ of the process states $x_{t}$ via the vector parameter $\rho =(\rho_{0},\rho_{1})\prime$, thus allowing to increase noise intensity with the magnitude of cell counts. Our proposed state-space formulation of Equations (7) and (8) can be interpreted as a hidden Markov model where all the states in ***x*** are latent, and some of these are measured as ***y*** through the measurement model of Equation (8).

### 2.3 Optimal filtering and smoothing

Consider the state-space model defined by Equations (7) and (8). Let $y_{1:\tau }$ be the vector of measurements collected at time $t=t_{1},t_{2},\ldots ,t_{\tau }$, and $x_{1:k}$ the process’ states from time *t*_1_ to time *t_k_*, where k=1,…,τ. Assuming the Markov properties


(9) $$\begin{array}{c} p(x_{k}|x_{1:k-1},y_{1:k-1};\theta )=p(x_{k}|x_{k-1};\theta )\\ p(x_{k-1}|x_{k:\tau },y_{k:\tau };\theta )=p(x_{k-1}|x_{k};\theta )\\ p(y_{k}|x_{1:k},y_{1:k-1};\rho )=p(y_{k}|x_{k};\rho ), \end{array}$$

for the distributions involved in the dynamic and measurement models of Equations (7) and (8), the aim of optimal filtering and smoothing is to estimate


(10) $$p(x_{k}|y_{1:\tau };\psi ),\;\psi =(\theta \prime ,\rho \prime )\prime ,$$

called predictive (k>τ), filtering (k=τ) and smoothing (k<τ) distributions, as a replacement of the (usually intractable) distribution $p(x_{0:\tau }|y_{1:\tau })$. Assuming a prior distribution $x_{0}∼N_{n}(x_{0}|m_{0},P_{0})$ for $x_{t}$ at *t *=* *0, the distributions of Equation (10) are Gaussian, whose first two moments, and the underlying vector parameter ψ, can be estimated by our proposed iterative algorithm which is summarized as follows (see Supplementary Section S6 for details):

1. **Prediction**: Solve the differential moment equations (DMEs)
  (11a) $$\{\begin{array}{c} \frac{dm^{*}(t)}{dt}=V_{\theta }m^{*}(t)\\ m^{*}(t_{k-1})=m_{k-1} \end{array}$$
   
  (11b) $$\{\begin{array}{c} \frac{dP^{*}(t)}{dt}=V_{\theta }P^{*}(t)+P^{*}(t)V\prime_{\theta }+\Delta t\beta (m^{*}(t),\theta )\\ P^{*}(t_{k-1})=P_{k-1} \end{array}$$
  to obtain the first two moments $m_{k}^{*}$ and $P_{k}^{*}$ of the predictive distribution at time *t_k_* (k=1,…,τ), where $V_{\theta }x$ is a re-formulation of Vh(x;θ) as a linear function of ***x***.
2. **Update**: Compute the first two moments $m_{k}$ and $P_{k}$ of the filtering distribution at time *t_k_* (k=1,…,τ) via the following correction step
  (12) $$\begin{array}{c} \mu_{k}=G_{k}m_{k}^{*},\\ S_{k}=G_{k}P_{k}^{*}G_{k}^{\prime }+R_{k},\\ K_{k}=P_{k}^{*}G_{k}^{\prime }S_{k}^{-1},\\ m_{k}=m_{k}^{*}+K_{k}(y_{k}-\mu_{k}),\\ P_{k}=P_{k}^{*}-K_{k}S_{k}K_{k}^{\prime }, \end{array}$$
  where $m_{k},\;P_{k},\;m_{k}^{*},\;P_{k}^{*},\;\mu_{k}$ and $S_{k}$ depend on ψ.
3. **Optimization**: Optimize the marginal likelihood of the measurements
  (13) $$\begin{array}{c} \psi \leftarrow \underset{\psi \geq 0}{\mathrm{argmin}}-\ell (\psi |y_{1},\ldots ,y_{\tau })\\ y_{k}∼N(\mu_{k}(\psi ),S_{k}(\psi )),\;\forall k=1,\ldots ,\tau . \end{array}$$
4. **Smoothing**: Estimate $x_{k}|y_{1:\tau }∼N(m_{k|\tau }^{s},P_{k|\tau }^{s}),\;k=1,\ldots ,\tau$, using the following backward step
  (14) $$\{\begin{array}{c} B_{k+1}=P_{k}e^{V\prime_{\theta }}{\left(P_{k+1}^{*}\right)}^{-1}\\ m_{k|\tau }^{s}=m_{k}+B_{k+1}(m_{k+1|\tau }^{s}-m_{k+1}^{*})\\ P_{k|\tau }^{s}=P_{k}+B_{k+1}(P_{k+1|\tau }^{s}-P_{k+1}^{*})B\prime_{k+1} \end{array}$$
  where $e^{\left(\cdot \right)}$ is the matrix exponential operator. We use a gradient-based method to solve the optimization problem of Equation (13). The gradient $\nabla_{\psi }\ell (\psi |y_{1},\ldots ,y_{\tau })$ of the marginal log-likelihood of the measurements is estimated numerically with *p *+* q* additional prediction and update steps, at time *t_k_*, k=1,…,τ, to compute all the partial derivatives of $\mu_{k}(\psi )$ and $S_{k}(\psi )$ w.r.t. ψ, where *p* and *q* are the dimensions of θ and ρ. Steps 1–4 are iterated until convergence is reached. The inference procedure is summarized in Supplementary Algorithm S2.

### 2.4 Transition probabilities

The transition probability *p_ij_* from cell type *i* to cell type *j* is defined as the multinomial probability


(15) $$\begin{array}{c} p_{ij}=\frac{\lambda_{ij}}{\alpha_{i}+\sum_{k\in O(i)}\lambda_{ik}}, \end{array}$$

where O(i) is the offspring set of cell type *i*, as defined by Equation (3).

### 2.5 Reaction constraints

To ensure identifiability of parameters in Equation (13) that involve only unobserved cell types, we use the following conservation laws


(16) $$\lambda_{ab}=\sum_{j}\lambda_{bc_{j}},\;c_{j}\in O(b),\;j=1,\ldots ,|O(b)|,$$

where O(b) is the offspring set of cell type *b*, as defined by Equation (3), and the linear constraints


(17) $$\begin{array}{c} \lambda_{a^{u}b^{u}}=\frac{1}{m}\sum_{j=1}^{m}\lambda_{a^{u}b_{j}^{o}},\alpha_{a^{u}}=\frac{1}{l}\sum_{j=1}^{l}\alpha_{a_{j}^{o}},\delta_{a^{u}}=\frac{1}{l}\sum_{j=1}^{l}\delta_{a_{j}^{o}},\\ a^{u}\in \chi^{u},\;b_{j}^{o}\in O(a^{u})\cap \chi^{o},\;a_{j}^{o}\in \chi^{o}\cap B(a^{u}),\\ A(a^{u})=A(a_{j}^{o}),\;|O(a^{u})\cap \chi^{o}|=m,\;|\chi^{o}\cap B(a^{u})|=l, \end{array}$$

where *χ^u^* and *χ^o^* are the sets of nonmeasured and measured cell types, B(x) represents the branch (Myeloid or Lymphoid) of cell lineage *x*, and finally A(x) is defined as the ancestor of cell type *x*. The constraint of Equation (16) can be viewed as a conservation law ensuring that all the cells differentiating from a cell lineage *b* to its offspring cells *c_j_*, j=1,…,|O(b)|, are those that were previously produced by an ancestor *a* of *b*. The first constraint of Equation (17) assumes that an unobserved cell type *a^u^* may differentiate into an unobserved cell type *b^u^* with a rate that equals the average rate of differentiation of *a^u^* into its observed offspring cells $b_{j}^{o},\;j=1,\ldots ,m$. The last two constraints of Equation (17) state that an unobserved cell type *a^u^* may duplicate (or die) with a rate that equals the average duplication (death) rate of the observed cells $a_{j}^{o},\;j=1,\ldots ,l$, belonging to the branch $B(a^{u})$ and sharing an ancestor with *a^u^*.

### 2.6 Haematopoietic models

**Figure 2.**
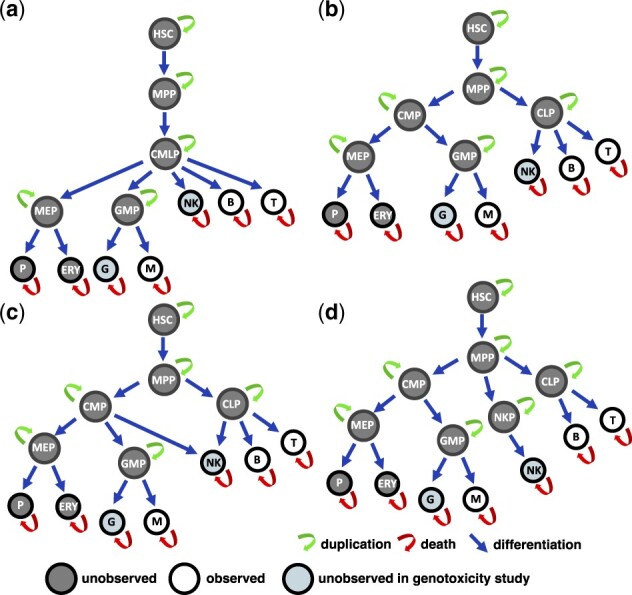
Graphical representation of the cell differentiation networks proposed as candidate models in the *in vivo* studies (a-d). Grey and white nodes represent unobserved and observed cell types. Light-grey nodes are the cell lineages whose data were collected in all studies except for the genotoxicity one. Arrows represent cell duplication (green), cell death (red), and cell differentiation (blue).

The candidate models of cell differentiation are shown in Fig. 2. The simplest model (a) is a single-branch developmental tree where the HSCs produce all the blood cells through a single multipotent progenitor (MPP) that first differentiate into common myeloid-lymphoid progenitors (CMLP) giving rise to the mature blood lymphoid cells (NK, B, and T) without any further intermediate progenitor. Whereas the erythroid cells (platelets P and erythrocytes ERY) and myeloid cells (granulocytes G and monocytes M) are produced via the Megakaryocyte–erythroid progenitor (MEP) cells and the impaired adult myeloid progenitor (GMP) cells respectively. According to model (b) the lymphoid cells (T, B, NK) and the myeloid/erythroid cells (G, M, P, ERY) are generated through separate branches of differentiation. In particular, MPP cells first differentiate into either common myeloid progenitor (CMP) cells or common lymphoid progenitor (CLP) cells, before giving rise to MEP and GMP cells. This model is known as classical/dichotomic model of haematopoiesis (Kawamoto and Katsura 2009). In contrast, model (c) proposes the idea that myeloid progenitors represent a prototype of haematopoietic cells capable to produce both myeloid (G, M) cells, erythroid (P, ERY) cells, and lymphoid NK cells, whereas lymphoid T, B, and NK cells represent specialized types produced by the CLP cells. Indeed, CMP cells not only produce MEP and GMP cells, but also NK cells. This model is known as the myeloid-based model (Kawamoto and Katsura 2009). Finally, model (d) assumes that lymphoid T/B cells and lymphoid NK cells develop through different branches. While B and T cells are produced by the common progenitor CLP, the NK cells are generated by a separate progenitor NKP.

### 2.7 Model selection

The candidate models of cell differentiation are scored according to the Akaike information criterion (AIC) (Burnham *et al.* 2011), i.e.


(18) $$\mathrm{AIC}(M)=2p_{M}-2\ell_{M}(\psi |y_{1},\ldots ,y_{\tau }),$$

where $\ell_{M}$ is the marginal log-likelihood of the measurements of model M and $p_{M}$ its number of parameters.

## 3 Results

**Figure 3.**
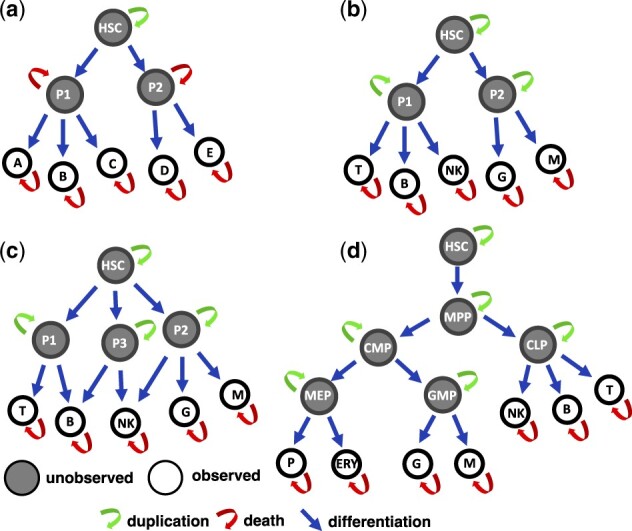
Graphical representation of the cell differentiation networks used in the in-silico studies (a-d). Grey and white nodes represent unobserved and observed cell types. Arrows represent cell duplication (green), cell death (red), and cell differentiation (blue).

We tested our proposed method in several simulation studies based on the validation scenarios from Fig. 3. These networks are sufficiently complex and diverse, in terms of number of nodes and depth, to assess the performance of our method against recovery of the true data generative process and underlying parameters. After validating our method, we analysed data from two preclinical studies and three gene therapy clinical trials to shed light on cell differentiation and haematopoietic reconstitution. The specific results are reported in the next subsections.

### 3.1 Validation and comparison with the prior art

We tested our proposed method Karen and compared it with the state-of-the-art approaches, such as the generalized least squares (GLS) method (Pellin *et al.* 2019), the maximum likelihood method RestoreNet (Del Core *et al.* 2023), and the branchCorr method (Xu *et al.* 2019). The validation and comparisons were made in terms of robustness against (i) the sampling frequency *τ*, (ii) the fraction *ζ* of false-negatives, and (iii) the magnitude of the measurement noise parameters *ρ*_0_ and *ρ*_1_. To make the comparison of our method with the other candidates possible, we used the cell differentiation structure of Fig. 3a as the true generative model, whose type of biochemical reactions is the only one that can be handled by Xu *et al.* (2019). The corresponding biochemical reactions and the system of SDEs were defined following Equations (1–6), using the state-space formulation of Equations (7) and (8). Two different comparative synthetic studies have been designed. In the first one all the cell types were measured, thus branchCorr was not included, since it does not allow for observed progenitors. In the second study the synthetic HSCs and progenitors P1–P2 were considered as unobserved states, and therefore GLS and RestoreNet were excluded from this comparison, since both methods do not allow for unobserved states. We used the Euler–Maruyama Algorithm S1 of the Supplementary Information to forward-simulate 100 independent stochastic trajectories of three clones from the true generative data process. Details on parameters and initial condition $x_{0}$ used for the simulations are reported in Supplementary Tables S1 and S2. Then we used our proposed framework Karen and the other methods to infer the unknown parameters. Inference with Karen has been carried out using Supplementary Algorithm S2, with and without the conservation laws of Equation (16) that ensure identifiability of the parameters involving only unobserved cell types.

**Figure 4.**
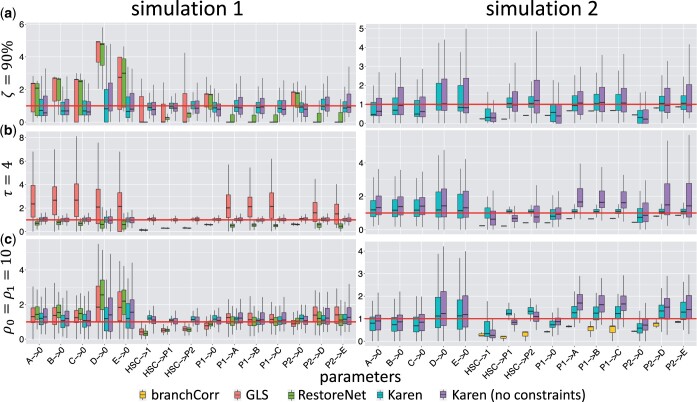
For each comparative synthetic study with observed (left) and systematically missing (right) progenitors HSC, P1, and P2: boxplots (*y*-axis) of the estimated parameters divided by the true ones for each reaction rate (*x*-axis) obtained from each method (colours), across all simulations, under a fraction ζ=90% of false negative errors (top), a sampling frequency *τ *= 4 (middle), and a measurement noise generated by $\rho_{0}=\rho_{1}=10$ (bottom).

Results from Fig. 4 clearly indicate that our proposed method outperformed the prior art. In particular, Fig. 4a provides evidence that our proposed method is the most robust with respect to the false negative errors compared to the other methods, which provided more biased estimates for the parameters for a high percentage ζ=90% of missing data. Subsequently, Fig. 4b shows that a low sampling frequency (*τ *= 4) of the simulated trajectories did not affect the estimates provided by our proposed method, whereas those obtained with any of the competitor approaches were biased. Finally, Fig. 4c suggests that after increasing the magnitude of the measurement noise parameters *ρ*_0_ and *ρ*_1_ up to 10, our proposed method still provided better estimates compared to the other methods. Further results for different values of *ζ*, *τ*, *ρ*_0_, and *ρ*_1_ can be found in Supplementary Section S7. In conclusion, the results from our synthetic studies show that overall our method outperformed the prior art in terms of false negative errors, sampling frequency and measurement noise. Details on the computational complexity are reported in Supplementary Section S10.

### 3.2 Model misspecification

**Figure 5.**
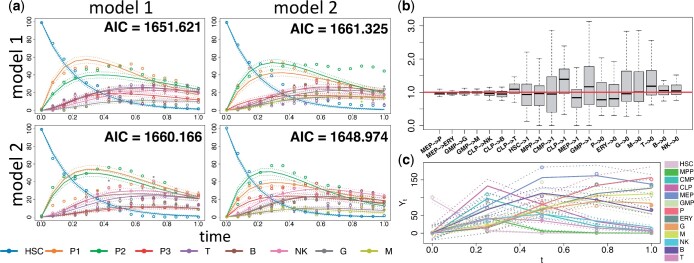
(a) For each true data generating process (row) and for each candidate model (column), the simulated process (empty dots), the synthetic data (full dots), the estimated smoothing moments (lines) of a single clone for each cell type (colours), and the median AIC across all simulations used to evaluate model misspecification. (b) Boxplots (*y*-axis) of the ratio between the estimated and true parameters for each reaction (*x*-axis) of the cell differentiation network of Fig. 3d used in the scalability study. (c) Estimated smoothing moments (lines), the true Markov states (empty dots), and the synthetic data (full dots) of one clone for each cell type (colours) for a single simulation of the cell differentiation network of Fig. 3d used in the scalability study.

We tested our method against model misspecification with an in-silico study. The cell differentiation networks of Fig. 3b and c were used as true generative models that we cross-compared for simulating and fitting. The corresponding biochemical reactions and the system of SDEs were defined following Equations (1–6), using the state-space formulation of Equations (7) and (8). We performed 100 independent simulations of the stochastic trajectories for three clones using the Euler–Maruyama Algorithm S1 of the Supplementary Information. Details on parameters and initial condition $x_{0}$ used for the simulations are reported in Supplementary Tables S3 and S4. Then we fitted both candidate models using the inference Supplementary Algorithm S2. Forward simulation and fitting has been carried out by using the conservation laws of Equation (16), so as to ensure identifiability of the parameters involving only unobserved cell types. As a result, Fig. 5a indicates that our method performed well in model selection, since the true models had the lowest median AIC, as defined by Equation (18), over 100 independent simulations. Moreover, Fig. 5a suggests that the true models yielded better fits in terms of the smoothing moments $m_{k|\tau }^{s}$ and $P_{k|\tau }^{s}$ (k=1,…,τ) compared to the wrong models.

### 3.3 Scalability to complex networks

We performed a synthetic study to explore the scalability of our proposed method to more complex network structures. We used the cell differentiation network of Fig. 3d as the true generative model. The corresponding biochemical reactions and the system of SDEs were defined following Equations (1–6), using the state-space formulation of Equations (7) and (8). As displayed in Fig. 3d, we assumed that the clonal cell counts were not collected from the HSCs and all the progenitors (MPP, CMP, CLP, MEP, GMP), and all the cell lineages that are missing for a particular clone at a given time point were also considered as unobserved. Therefore, for the measurement model of Equation (8) the selection matrix $G_{t}$ was defined accordingly. We performed 50 independent simulations of stochastic trajectories for 100 clones from the generative data process of Fig. 3d using the Euler–Maruyama Algorithm S1 of the Supplementary Information. Details on parameters and initial condition $x_{0}$ used for the simulations are reported in Supplementary Tables S5 and S6. Then we applied our inference method Karen on the simulated data using Supplementary Algorithm S2. Each simulation and fitting has been carried out by assuming the conservation laws of Equation (16), so as to ensure identifiability of the parameters that involve only unobserved cell types. Results from Fig. 5b and c show low uncertainty of the estimated parameters and a good recovery of the Markov states in terms of the first two smoothing moments $m_{k|\tau }^{s}$ and $P_{k|\tau }^{s},\;k=1,\ldots ,\tau$.

### 3.4 Genotoxicity study

**Figure 6.**
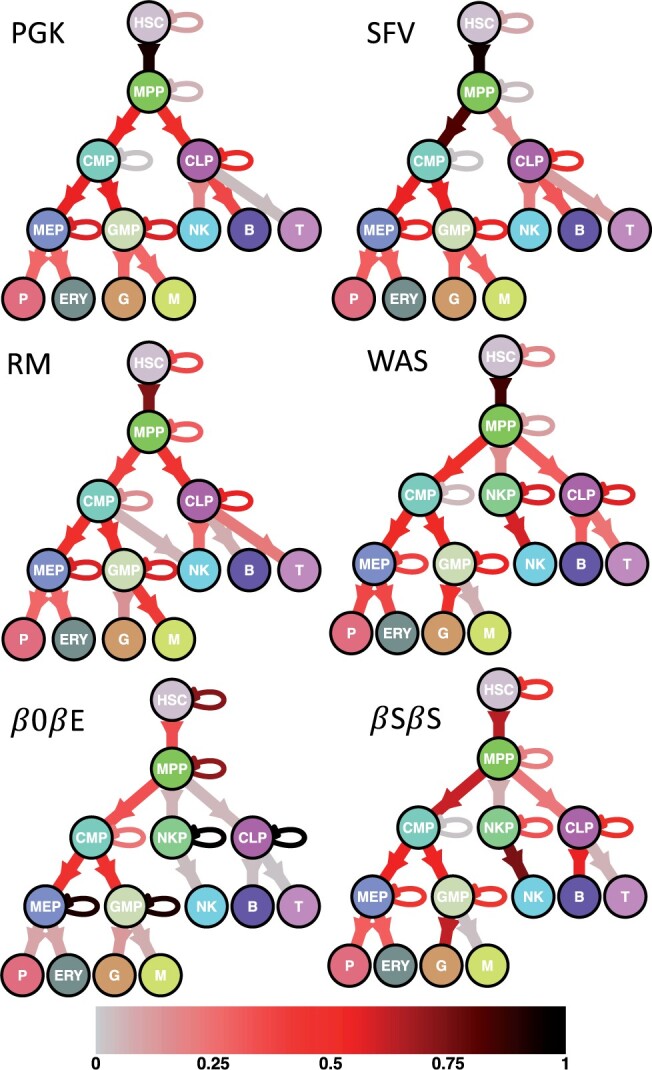
Inferred cell differentiation networks having the lowest AIC, as defined by Equation (18), for the genotoxicity study for the comparison of the two viral vectors PGK and SFV, the rhesus macaque (RM) study, and the clinical trials WAS, *β*0*β*E and *β*S*β*S. Each arrow is weighted and coloured according to the transition probabilities estimated with Equation (15).

We analysed an *in vivo* clonal tracking dataset previously used to investigate the impact of vector design on clonal diversity in tumour-prone mice (Del Core *et al.* 2022). Cdkn2a${}^{-/-}$ tumour prone Lin${}^{-}$ cells were first *ex vivo* transduced with a lentiviral vector expressing GFP under either spleen focus-forming virus (SFV) or PGK promoter/enhancer sequence. Cells are then transplanted into lethally irradiated wild-type mice. To recover enough DNA material, equal amounts of blood from two or three mice belonging to the same experimental group were pooled before cell sorting. Integration sites were then retrieved by polymerase chain reaction (PCR) at different time points from sorted T (CD3+) and B (CD19+) lymphocytes and myeloid cells (CD11b+). Clonal tracking samples were collected under heterogeneous technical conditions (see Supplementary Table S10), making them not directly comparable. Therefore, we rescaled the data following the description in Supplementary Section S11. The total number of distinct clones that were collected are 45 186 and 20 471 for the PGK and SFV treatments, respectively. To further remove bias, we focused our analyses on the top 1000 most recaptured clones across lineages and time. We used the inference Supplementary Algorithm S2 to fit the four biologically sustained models of cell differentiation from Fig. 2 under the two vector conditions PGK and SFV. For each model the system of SDEs was defined following Equations (1–6), using the state-space formulation of Equations (7) and (8). Inference has been carried out by assuming the conservation laws of Equations (16) and (17), so as to ensure identifiability of the parameters involving only systematically unobserved cell types. For each candidate model we computed the Akaike information criterion (AIC), as defined by Equation (18), and we report the results on model selection in Table 1. According to the AIC, model (b) is the one that best fitted clonal tracking data under each vector design. The corresponding cell differentiation networks are displayed in Fig. 6. This result suggests that the classical/dichotomic model structure (b) adequately described clonal dynamics in tumour-prone mice under both treatments. Also, the arrow weights from Fig. 6 clearly indicate that in SFV-treated tumour-prone mice there was a more pronounced unbalance in cell differentiation from the multipotent progenitors (MPP) towards common myeloid progenitors (CMP) compared to the PGK treatment. Therefore our proposed framework Karen suggests that, in this particular study, the design of viral vector did not significantly affect the structure of cell differentiation in tumour-prone mice, but had an impact on the transition probabilities p(MPP→CMP) and p(MPP→CLP), representing differentiation from the multipotent progenitors compartment (MPP) in either common myeloid (CMP) or lymphoid (CLP) progenitors.

**Table 1.** For each *in vivo* clonal tracking dataset analysed (rows) and the candidate models a–d (columns) the AIC computed according to Equation (18).

	a	b	c	d
PGK	128 230.94	120 429.29	120 454.55	323 765.06
LTR	76 220.33	75 102.02	75 206.44	75 161.76
RM	138 921.13	802 760.11	108 993.15	109 578.07
WAS	103 931.52	102 959.02	508 888.96	102 444.74
*β*0*β*E	50 900.45	50 142.89	50 936.42	49 960.66
*β*S*β*S	46 665.12	45 876.46	45 898.89	45 872.07

### 3.5 Rhesus Macaques study

We analysed an *in vivo* clonal tracking dataset collected from Rhesus Macaques (Wu *et al.* 2014). HSCs were first barcoded by using lentiviral vectors and then transplanted in three animals. Barcode retrieval was performed monthly via PCR on Granulocytes (G), Monocytes (M), T, B, and NK cells up to 9.5 months. Further details on transductions protocol and culture conditions can be found in Wu *et al.* (2014). Although the sample DNA amount was maintained constant during the whole experiment, the samples resulted in different magnitudes of reads (see Supplementary Table S11), making the data not directly comparable. Therefore, we rescaled the barcode counts as described in Supplementary Section S12 before analysis. The total numbers of clones that were collected range in 1165–1291, but we focused on the top 1000 most recaptured ones, across lineages and time, so as to further remove bias. We fitted the same four candidate models from the previous section on the clonal tracking data using Supplementary Algorithm S2. For each model the system of SDEs was defined following Equations (1–6), using the state-space formulation of Equations (7) and (8). In analogy with the previous section, inference has been performed by using the conservation laws of Equations (16) and (17) that ensure identifiability of the parameters involving only unobserved cell lineages. Each candidate model has been scored according to the Akaike information criterion (AIC) of Equation (18) and we report the results on model selection in Table 1. According to the AIC, model (c) is the one that best fitted the clonal tracking data collected from the rhesus macaque study. The estimated cell differentiation network is reported in Fig. 6. This result suggests that the classical/dichotomic model (b) failed to describe adequately clonal dynamics in rhesus macaques, whereas the myeloid-based developmental model (c) better explained haematopoietic reconstitution. Our proposed framework Karen clearly indicates that myeloid progenitors represent a prototype of haematopoietic cells capable to produce both myeloid (G, M) cells, erythroid (P, ERY) cells and lymphoid NK cells in primates.

### 3.6 Gene therapy clinical trials

We considered clonal tracking data collected from six patients affected by three different genetic disorders. The six patients underwent a haematopoietic stem and progenitor cell (HSPC) gene therapy treatment. Vector integration sites in five cell lineages (G, M, T, B, and NK) were collected longitudinally from the peripheral blood of four patients affected by Wiskott–Aldrich syndrome (WAS) (Hacein-Bey Abina *et al.* 2015), two patients with *β* haemoglobinopathy, one with *β*S/*β*S sickle cell disease (Ribeil *et al.* 2017), and one with *β*0/*β*E *β* thalassaemia (Thompson *et al.* 2018). Details on procedures, gene therapy protocols, and normalization methods can be found in Hacein-Bey Abina *et al.* (2015), Ribeil *et al.* (2017), and Thompson *et al.* (2018). Since data were already normalized to compensate for unbalanced sampling in VCN and DNA (Sherman *et al.* 2017), we did not apply any further transformation. The total clones that were collected are 156 654, 17 273, and 230 408, respectively, for WAS, *β*S/*β*S, and *β*0/*β*E clinical trials. The following results stem from the analysis of the 1000 most recaptured clones, across lineages and time, in each clinical trial. The four haematopoietic models of Fig. 2 have been scored separately in each clinical trial using Supplementary Algorithm S2. For each model the system of SDEs was defined following Equations (1–6), using the state-space formulation of Equations (7) and (8). In analogy with previous sections, inference has been carried out by assuming the linear constraints of Equations (16) and (17) ensuring identifiability of the parameters that involve only the unobserved cell types. For each candidate model we computed the Akaike information criterion (AIC), as defined by Equation (18), and we report the results on model selection in Table 1. According to the AIC, model (d) is the one that best fitted clonal tracking data collected from each clinical trial, and the corresponding cell differentiation networks are reported in Fig. 6. Results suggest that a three-branches developmental model best explained haematopoietic reconstitution in these gene therapy clinical trials. While lymphoid (T, B) and myeloid/erythroid cells (G, M, P, ERY) developed in parallel through separate branches from different progenitors, NK cells appear to be sustained by a dedicated progenitors’ cell population.

## 4 Discussion

We have proposed a novel stochastic framework for calibrating cell differentiation networks from partially observed high-dimensional clonal tracking data. Our model is able to deal with experimental clonal tracking data that suffers from measurement noise and low levels of clonal recapture due to either threshold detection failures or false-negative errors. Our proposed framework Karen extends stochastic quasi-reaction networks by introducing an EKF and an RTS smoother. We have developed an E–M algorithm to infer the corresponding parameters. Simulation studies have shown the method’s accuracy regarding inference of the true parameters, estimation of the first two smoothing moments of all the process states, and model selection using AIC. Simulation results indicated higher robustness of our proposed method compared to the state-of-the-art ones in terms of (i) low sampling frequency, (ii) limited clonal recapture, and (iii) high levels of measurement noise. Results from simulations suggest that our proposed framework scales to complex structures of cell differentiations in terms of nodes size and network depth. Although the Gaussian assumption makes the analytical formulations of the likelihoods explicitly available, this approximation may become poor when the data contains outliers or shows non-Gaussian behaviours. This limitation can be overcome by using a distribution-free approach, such as the Kernel Kalman Rule, a recent nonparametric inference technique for high dimensional and possibly non-Gaussian nonlinear state-space models (Gebhardt *et al.* 2019). Besides, our framework considers reaction rates constant for the whole study period. Extensions that allow for modelling reaction rates as smooth functions of time or clinically relevant variables will be the goal of our future research.

Our proposed method allowed to unveil the genotoxic impact on cell differentiation in tumour-prone mice. While the differentiation structure does not seem to be affected by the viral vector design, the transition probabilities from the multipotent progenitors to the intermediate progenitors do, showing a more pronounced unbalance towards the common myeloid progenitors under the SFV treatment compared to PGK. This can be biologically interpreted as a faster immune response to the higher inflammation caused by the toxic SFV treatment, compared to the nontoxic PGK one. Subsequently, the application of Karen to a rhesus macaque clonal tracking study unveiled for the lymphoid NK cells a different developmental pathway from the one detected for lymphoid T and B cells. That is, NK cells are produced by both common myeloid CMP and lymphoid CLP progenitors, whereas T and B cells are sustained only by the common lymphoid progenitors CLP. Results are consistent with those previously reported in Wu *et al.* (2014), where the authors demonstrated the presence of distinct subpopulations within the NK cells lineage, potentially deriving from alternative maturation processes. Finally, it is worth noting the agreement in the inferred network structure between the different clinical trials. Our modelling approach is able to capture the heterogeneity in cell repopulation dynamics and selective advantage of different contexts, as suggested by the parameter estimates. Our stochastic framework can support biologists to shed light on haematopoietic reconstitution and in designing tailor-made therapies to treat genetic disorders. Our model can be applied to different types of clonal tracking data, such as vector integration sites, clonal barcodes, and single cell methods. Applications in alternative contexts of population dynamics, showing similar issues of partial sampling and measurement noise, could also be explored.

## Supplementary Material

btad605_Supplementary_Data
