# Supplementary material for: Scalable inference of cell differentiation networks in gene therapy clonal tracking studies of haematopoiesis
Source: Bioinformatics. 2023 Sep 29;39(10):btad605. doi: 10.1093/bioinformatics/btad605 (PMC10585354; doi:10.1093/bioinformatics/btad605)
Supplement: btad605_Supplementary_Data [file btad605_supplementary_data.pdf]

Scalable inference of cell differentiation networks in gene therapy clonal tracking studies of haematopoiesis

Supplementary Information

L. Del Core et al.
luca.delcore@nottingham.ac.uk

Contents

S.1	Stochastic quasi-reaction networks	2
S.2	The Master Equation	3
S.3	Euler-Maruyama simulation of Itô-type SDEs	4
S.4	Differential Sylvester Equation	4
S.5	Integrating factor method	5
S.6	Kalman reaction networks (Karen)	5
S.7	Validation and comparison with the prior art	9
S.8	Model misspecification	10
S.9	Scalability to complex networks	10
S.10	Computational complexity	12
S.11	Genotoxicity data rescaling	13
S.12	Rhesus macaque data rescaling	14

Algorithms

Algorithm S.1	Euler-Maruyama simulation scheme	4
Algorithm S.2	Inference procedure	9

Tables and Figures

Table S.1:	Validation study - dynamic vector parameter	9
Table S.2:	Validation study - simulation parameters	10
Figure S.1:	Validation study - results	11
Table S.3:	Model misspecification - dynamic vector parameter	12
Table S.4:	Model misspecification - simulation parameters	12
Table S.5:	Scalability study - dynamic vector parameter	12
Table S.6:	Scalability study - simulation parameters	12
Table S.7:	Computational complexity - dynamic vector parameter	13
Table S.8:	Computational complexity - simulation parameters	13
Table S.9:	Computational complexity - results	13
Table S.10:	Genotoxicity study - confounders	14
Table S.11:	Rhesus macaque study - number of reads	15

S.1 Stochastic quasi-reaction networks

Stochastic quasi-reaction networks (S-QRNs) are a particular class of stochastic differential equations suitable to model biochemical reactions. More formally, let

$$\boldsymbol{y}_t = (y_{1t}, \dots, y_{nt})' \in \mathbb{N}_0^n \tag{1}$$

be a collection of molecules of n different types observed at time t, and consider K distinct (and competing) reactions

$$r_{j1}y_1 + \cdots + r_{jn}y_n \xrightarrow{\theta_j} p_{j1}y_1 + \cdots + p_{jn}y_n \,, \quad j = 1, \dots, K \,, \tag{2}$$

each occurring with its own rate θ_j. The coefficients r_{ji}'s defining the left-side of the reaction are called reagents and represent the minimum amount of molecules of type i needed for the j-th reaction to occur. Similarly, the coefficients p_{ji} defining the right-side of the reaction are called products and represent the amount of produced molecules of type i after the j-th reaction is triggered. We assume that, if we observe $\boldsymbol{y}_0 = (r_{j1}, \dots, r_{jn})'$ molecules at time $t = 0$, the j-th reaction will occur after

$$T_j \sim \text{Exp}(\theta_j) \,, \quad j = 1, \dots, K \,, \tag{3}$$

Namely, if exactly r_{ij} molecules of each type i would be present, then the j-th reaction can only take place in one way, with the exponential hazard rate θ_j. The interpretation is that, after a waiting time T_j, r_{ji} molecules of type i collide with each other and produce p_{ji} molecules of type i ($\forall i = 1, \dots, n$), while the molecules move randomly in a hosting "cellular" environment. However, in general at time $t = 0$ we might observe $y_{i0} \geq r_{ji}$ molecules of each type i and, therefore, the j-th reaction can take place in a combinatorial number of ways leading to the following waiting time formulation

$$T_j \sim \text{Exp}\left(\theta_j \prod_{i=1}^{n} \binom{y_{i0}}{r_{ji}}\right) \,, \quad \text{where } \binom{x}{y} = 0 \,, \quad \text{for } x < y \,, \tag{4}$$

where

$$\boldsymbol{\theta} = (\theta_1, \dots, \theta_K)' \tag{5}$$

is the vector parameter for the reaction rates, and

$$h_j(\boldsymbol{y}_0, \boldsymbol{\theta}) = \theta_j \prod_{i=1}^{n} \binom{y_{i0}}{r_{ji}} \tag{6}$$

is the j-th hazard rate. In this case, the effect will be that at time $t + T_j$ we have the following expression for the number of molecules of substrate i,

$$y_{i,t+T_j} = y_{it} + p_{ji} - r_{ji} = y_{it} + v_{ji} \,, \tag{7}$$

where $v_{ji} = p_{ji} - r_{ji}$ is the j-th net effect. More compactly, for a set of K reactions and n species, the molecular transfer from reagent to product species is a net change of

$$\boldsymbol{V} = \boldsymbol{P} - \boldsymbol{R} \,, \tag{8}$$

where $\boldsymbol{P} = [p_{ji}]'$ denotes the $n \times r$ dimensional matrix of products, $\boldsymbol{R} = [r_{ji}]'$ is the $n \times r$ dimensional matrix of reactants, and $\boldsymbol{V} = [v_{ji}]'$ is an $n \times r$ dimensional matrix called net-effect matrix. Therefore, a S-QRN of K-distinct reactions is fully identified by a net-effect matrix $\boldsymbol{V}$ and by the hazard vector

$$\boldsymbol{h}(\boldsymbol{y}, \boldsymbol{\theta}) = (h_1(\boldsymbol{y}, \boldsymbol{\theta}), \ldots, h_K(\boldsymbol{y}, \boldsymbol{\theta}))' \; . \tag{9}$$

S.2 The Master Equation

In practice it is common that the reaction rates of a stochastic reaction network are unknown, and the goal is to estimate them given a collected dataset. In order to estimate the rates $\boldsymbol{\theta} = (\theta_1, \ldots, \theta_K)'$ using a likelihood-based approach, we need to define an underlying probabilistic model. One of the most natural choices for describing stochastic chemical kinetics of Eqs. (2-9) is the chemical master equation

$$\frac{dP(\boldsymbol{y};t)}{dt} = \sum_{j=1}^{K} \{h_j(\boldsymbol{y} - V_{\cdot j}; \boldsymbol{\theta})P(\boldsymbol{y} - V_{\cdot j}; t) - h_j(\boldsymbol{y}; \boldsymbol{\theta})P(\boldsymbol{y}; t)\} \; , \tag{10}$$

with transition rates

$$h_j(\boldsymbol{y}; \theta) = \theta_j \prod_{i=1}^{n} \binom{y_i}{r_{ji}} \; , \tag{11}$$

consistently with Eq. (4). It describes the temporal evolution of the probability density function $P(\boldsymbol{y}; t)$ of the state vector $\boldsymbol{y}$ of the chemical system defined by Eq. (2). Roughly speaking, the first part of the right-hand side of Eq. (10) models all the reactions letting the state out of $k(\neq j)$, whereas the second part models all the reactions which brings the state back to k. It is often the case that the Master equation is computationally intractable, especially when the state vector $\boldsymbol{y}$ is high-dimensional, so that the number of possible states the system may occupy is too large. Several approximations of the Master equation exist (Érdi and Tóth, 1989; Sjöberg et al., 2009), and here we describe a procedure for "continuizing" the discrete-state chemical Markov process defined by Eqs. (2-10). The procedure is summarized in the following theorem.

Theorem 1. *Assume that $h_j(\boldsymbol{x}; \boldsymbol{\theta})P(\boldsymbol{x}; t)$ are analytical functions in $\boldsymbol{x}$. Then, a second order Taylor expansion of the products $h_j(\boldsymbol{x} - V_{\cdot j}; \boldsymbol{\theta})P(\boldsymbol{x} - V_{\cdot j}; t)$ around $\boldsymbol{x}$ leads to the Itô-type stochastic differential equation*

$$d\boldsymbol{x}_t = \mu(\boldsymbol{x}_t; \boldsymbol{\theta})dt + \beta^{1/2}(\boldsymbol{x}_t; \boldsymbol{\theta})d\boldsymbol{W}(t) \, , \quad d\boldsymbol{W}(t) \sim N(\boldsymbol{0}, dt\boldsymbol{I}) \, , \tag{12}$$

called the Kramers-Moyal approximation where the drift function and the dispersion matrix are given by

$$\boldsymbol{\mu}(\boldsymbol{x}_t; \boldsymbol{\theta}) = V\boldsymbol{h}(\boldsymbol{x}_t, \boldsymbol{\theta}) \, , \quad \boldsymbol{\beta}(\boldsymbol{x}_t; \boldsymbol{\theta}) = V \underbrace{\begin{bmatrix} h_1(\boldsymbol{x}_t; \boldsymbol{\theta}) & & \\ & \ddots & \\ & & h_K(\boldsymbol{x}_t; \boldsymbol{\theta}) \end{bmatrix}}_{d(\boldsymbol{h}(\boldsymbol{x}_t, \theta))} V' \, . \tag{13}$$

Input: $\boldsymbol{x}_0$, $\Pi_\tau = \{0 = t_0 < t_1 < \cdots < t_{\tau+1} = T\}$, $t_{k+1} - t_k = \Delta t$
Output: $\{\boldsymbol{x}_k\}_k$
for $k = 1 : \tau$ **do**
1. Draw ΔW_k from $\Delta W_k \sim N(0, \Delta t)$, $\quad t_k = k\Delta t$
2. Compute $\boldsymbol{x}_{k+1} = \boldsymbol{x}_k + \boldsymbol{\mu}(\boldsymbol{x}_k; \boldsymbol{\theta})\Delta t + \boldsymbol{\beta}(\boldsymbol{x}_k; \boldsymbol{\theta})\Delta W_k$

end

Algorithm S.1: Pseudocode of the Euler-Maruyama simulation scheme.

Proof. The analytical assumption of $h_j(\boldsymbol{x};\boldsymbol{\theta})P(\boldsymbol{y};t)$ in $\boldsymbol{x}$ allows us to consider a second-order Taylor expansion of $h_j(\boldsymbol{x} - V_{\cdot j};\boldsymbol{\theta})P(\boldsymbol{x} - V_{\cdot j};t)$ around $\boldsymbol{x}$, that is

$$\begin{aligned} & h_j(\boldsymbol{x} - V_{\cdot j};\boldsymbol{\theta})P(\boldsymbol{x} - V_{\cdot j};t) \\ & = h_j(\boldsymbol{x};\boldsymbol{\theta})P(\boldsymbol{x};t) + \nabla_{\boldsymbol{x}} h_j(\boldsymbol{x};\boldsymbol{\theta})P(\boldsymbol{x};t)\left((\boldsymbol{x} - V_{\cdot j}) - \boldsymbol{x}\right) \\ & +\frac{1}{2}\left((\boldsymbol{x} - V_{\cdot j}) - \boldsymbol{x}\right)' H_{\boldsymbol{x}} h_j(\boldsymbol{x};\boldsymbol{\theta})P(\boldsymbol{x};t)\left((\boldsymbol{x} - V_{\cdot j}) - \boldsymbol{x}\right) \\ = {} & h_j(\boldsymbol{x};\boldsymbol{\theta})P(\boldsymbol{x};t) - \nabla_{\boldsymbol{x}}\left\{h_j(\boldsymbol{x};\boldsymbol{\theta})P(\boldsymbol{x};t)\right\} V_{\cdot j} + \frac{1}{2}V'_{\cdot j} H_{\boldsymbol{x}}\left\{h_j(\boldsymbol{x};\boldsymbol{\theta})P(\boldsymbol{x};t)\right\} V_{\cdot j}\,, \end{aligned}$$

and therefore

$$\begin{aligned} & h_j(\boldsymbol{x} - V_{\cdot j};\boldsymbol{\theta})P(\boldsymbol{x} - V_{\cdot j};t) - h_j(\boldsymbol{x};\boldsymbol{\theta})P(\boldsymbol{x};t) \\ & = -\nabla_{\boldsymbol{x}}\left\{h_j(\boldsymbol{x};\boldsymbol{\theta})P(\boldsymbol{x};t)\right\} V_{\cdot j} + \frac{1}{2}V'_{\cdot j} H_{\boldsymbol{x}}\left\{h_j(\boldsymbol{x};\boldsymbol{\theta})P(\boldsymbol{x};t)\right\} V_{\cdot j}\,, \end{aligned}$$

and by plugging it in the Master Eq. (10) we have

$$\begin{aligned} \frac{\partial P(\boldsymbol{x},t)}{\partial t} & = \sum_{j=1}^{K}\left\{-\nabla_{\boldsymbol{x}}\left\{h_j(\boldsymbol{x};\boldsymbol{\theta})P(\boldsymbol{x};t)\right\} V_{\cdot j} + \frac{1}{2}V'_{\cdot j} H_{\boldsymbol{x}}\left\{h_j(\boldsymbol{x};\boldsymbol{\theta})P(\boldsymbol{x};t)\right\} V_{\cdot j}\right\} \\ & = -\nabla_{\boldsymbol{x}}\left\{\boldsymbol{V}\boldsymbol{h}(\boldsymbol{x};\boldsymbol{\theta})P(\boldsymbol{x};t)\right\} + \frac{1}{2}\nabla^2_{\boldsymbol{x}}\left\{\boldsymbol{V}\begin{bmatrix} h_1(\boldsymbol{x}_t;\boldsymbol{\theta}) & & \\ & \ddots & \\ & & h_K(\boldsymbol{x}_t;\boldsymbol{\theta}) \end{bmatrix}\boldsymbol{V}' P(\boldsymbol{x};t)\right\}\,, \end{aligned}$$

which we recognize as a Kolmogorov forward (Fokker-Plank) equation with drift function $\boldsymbol{V}\boldsymbol{h}(\boldsymbol{x};\boldsymbol{\theta})$ and dispersion matrix $\boldsymbol{V}d(\boldsymbol{h}(\boldsymbol{x};\boldsymbol{\theta}))\boldsymbol{V}'$, which completes the proof. □

S.3 Euler-Maruyama simulation of Itô-type SDEs

We simulate clonal trajectories from the system of Itô-type SDEs, as defined by Eqs. (12-13), using the Euler-Maruyama method (Kloeden and Platen, 2011) reported in Algorithm S.1.

S.4 Differential Sylvester Equation

Theorem 2. *Let $I \subseteq \mathbb{R}$ be an open interval with $t_0 \in I$ and $\boldsymbol{X}(t) \in \mathbb{R}^{n\times m}$, $\boldsymbol{A} \in \mathbb{R}^{n\times n}$, $\boldsymbol{B} \in \mathbb{R}^{m\times m}$, $\boldsymbol{C}(t) \in \mathbb{R}^{n\times m}$, and $\boldsymbol{D} \in \mathbb{R}^{n\times m}$. The differential Sylvester equation*

$$\begin{aligned} \dot{\boldsymbol{X}}(t) & = \boldsymbol{A}\boldsymbol{X}(t) + \boldsymbol{X}(t)\boldsymbol{B} + \boldsymbol{C}(t) \\ \boldsymbol{X}(t_0) & = \boldsymbol{D} \end{aligned} \tag{14}$$

has the unique solution (Behr et al., 2019)

$$\boldsymbol{X}(t) = e^{\boldsymbol{A}(t-t_0)}\boldsymbol{D}e^{\boldsymbol{B}(t-t_0)} + \int_{t_0}^{t} e^{\boldsymbol{A}(t-s)}\boldsymbol{C}(s)e^{\boldsymbol{B}(t-s)}ds\,. \tag{15}$$

S.5 Integrating factor method

A system of first order differential equations in standard form

$$\dot{\boldsymbol{y}} + \boldsymbol{A}(t)\boldsymbol{y} = \boldsymbol{b}\,, \tag{16}$$

where $\boldsymbol{y} \in \mathbb{R}^n$, and $\boldsymbol{A}(t) \in \mathbb{R}^{n\times n}$ and $\boldsymbol{b} \in \mathbb{R}^n$ has an explicit solution given by

$$\boldsymbol{y} = e^{-\int \boldsymbol{A}(t)dt}\left\{\int e^{\int \boldsymbol{A}(t)dt}\boldsymbol{b}dt + \boldsymbol{C}\right\}\,, \quad I = e^{\int \boldsymbol{A}(t)dt}\,, \tag{17}$$

where I is the integrating factor (Adams et al., 2021).

S.6 Kalman reaction networks (Karen)

We consider a continuous-discrete state space-model (CD-SSM) whose dynamic component is the system of Itô-type SDEs

$$d\boldsymbol{x} = \boldsymbol{\mu}(\boldsymbol{x};\boldsymbol{\theta})dt + \boldsymbol{\beta}(\boldsymbol{x};\boldsymbol{\theta})^{1/2}d\boldsymbol{W}\,, \quad d\boldsymbol{W} \sim \mathcal{N}_n(\boldsymbol{0}, dt\boldsymbol{I}_n)\,, \tag{18}$$

where $\boldsymbol{\mu}(\boldsymbol{x};\boldsymbol{\theta})$ and $\boldsymbol{\beta}(\boldsymbol{x};\boldsymbol{\theta})$ are defined by Eq. (13), combined with the measurement model

$$\begin{aligned} \boldsymbol{y}_t &= \boldsymbol{g}_t(\boldsymbol{x}_t, \boldsymbol{R}_t) = \boldsymbol{G}_t\boldsymbol{x}_t + \boldsymbol{r}_t; \quad \boldsymbol{r}_t \sim \mathcal{N}_d(\boldsymbol{0}, \boldsymbol{R}_t)\,, \\ \boldsymbol{R}_t &= \rho_0\boldsymbol{I}_d + \rho_1 \begin{bmatrix} (\boldsymbol{G}_k\boldsymbol{x}_t)_1 & & \\ & \ddots & \\ & & (\boldsymbol{G}_k\boldsymbol{x}_t)_d \end{bmatrix}\,, \end{aligned} \tag{19}$$

where $\boldsymbol{G}_t$ is a $d \times n$ time-dependent matrix selecting only the measurable states of $\boldsymbol{x}_t$ subject to an additive noise $\boldsymbol{r}_t$, and $\boldsymbol{x}_t$ is a shorthand notation for $\boldsymbol{x}(t)$. The covariance matrix $\boldsymbol{R}_t$ models the measurement noise as a linear function $\boldsymbol{G}_t\boldsymbol{x}_t$ of the process states $\boldsymbol{x}_t$ through the vector parameter $\boldsymbol{\rho} = (\rho_0, \rho_1)'$, thus allowing to increase noise intensity with the magnitude of cell counts. Our proposed state-space formulation of Eqs. (18-19) can be viewed as a stochastic hidden Markov model where all the states in $\boldsymbol{x}$ are latent, and some of these are measured as $\boldsymbol{y}$ through the measurement model of Eq. (19).

Let $\boldsymbol{y}_{1:\tau}$ be the vector of measurements collected at time $t = t_1, t_2, \ldots, t_\tau$, and $\boldsymbol{x}_{1:k}$ the process' states from time t_1 to time t_k, where $k = 1, \ldots, \tau$. Assuming the Markov properties

$$\begin{aligned} p(\boldsymbol{x}_k|\boldsymbol{x}_{1:k-1}, \boldsymbol{y}_{1:k-1}; \boldsymbol{\theta}) &= p(\boldsymbol{x}_k|\boldsymbol{x}_{k-1}; \boldsymbol{\theta}) \\ p(\boldsymbol{x}_{k-1}|\boldsymbol{x}_{k:\tau}, \boldsymbol{y}_{k:\tau}; \boldsymbol{\theta}) &= p(\boldsymbol{x}_{k-1}|\boldsymbol{x}_k; \boldsymbol{\theta}) \\ p(\boldsymbol{y}_k|\boldsymbol{x}_{1:k}, \boldsymbol{y}_{1:k-1}; \boldsymbol{\rho}) &= p(\boldsymbol{y}_k|\boldsymbol{x}_k; \boldsymbol{\rho})\,, \end{aligned} \tag{20}$$

the aim of optimal filtering and smoothing is to estimate

$$p(\boldsymbol{x}_k|\boldsymbol{y}_{1:\tau}; \boldsymbol{\psi})\,, \quad \boldsymbol{\psi} = (\boldsymbol{\theta}', \boldsymbol{\rho}')'\,, \tag{21}$$

called predictive ($k > \tau$), filtering ($k = \tau$) and smoothing ($k < \tau$) distributions, as a replacement of the (usually intractable) distribution $p(\boldsymbol{x}_{0:\tau}|\boldsymbol{y}_{1:\tau})$. Assuming a prior distribution $\boldsymbol{x}_0 \sim \mathcal{N}_n(\boldsymbol{x}_0|\boldsymbol{m}_0, \boldsymbol{P}_0)$ for $\boldsymbol{x}_t$ at $t = 0$, the distributions of Eq. (21) are Gaussian, whose first two moments, and the underlying vector parameter $\boldsymbol{\psi}$, can be estimated by our proposed iterative algorithm which is summarised as follows.

1. Prediction: Solve the differential moment equations (DMEs)

$$\begin{cases} \frac{d\boldsymbol{m}^*(t)}{dt} = \boldsymbol{V}_{\boldsymbol{\theta}}\boldsymbol{m}^*(t) \\ \boldsymbol{m}^*(t_{k-1}) = \boldsymbol{m}_{k-1} \end{cases} \tag{22a}$$

$$\begin{cases} \frac{d\boldsymbol{P}^*(t)}{dt} = \boldsymbol{V}_{\boldsymbol{\theta}}\boldsymbol{P}^*(t) + \boldsymbol{P}^*(t)\boldsymbol{V}'_{\boldsymbol{\theta}} + \Delta t\boldsymbol{\beta}(\boldsymbol{m}^*(t), \boldsymbol{\theta}) \\ \boldsymbol{P}^*(t_{k-1}) = \boldsymbol{P}_{k-1} \end{cases} \tag{22b}$$

to obtain the first two moments $\boldsymbol{m}^*_k$ and $\boldsymbol{P}^*_k$ of the predictive distribution at time t_k ($k = 1, \ldots, \tau$), where $\boldsymbol{V}_{\boldsymbol{\theta}}\boldsymbol{x}$ is a re-formulation of $\boldsymbol{V}\boldsymbol{h}(\boldsymbol{x}; \boldsymbol{\theta})$ as a linear function of $\boldsymbol{x}$. The solutions of Eqs. (22a-22b) are given by

$$\boldsymbol{m}^*(t) = e^{\boldsymbol{V}_{\boldsymbol{\theta}}(t-t_{k-1})}\boldsymbol{m}_{k-1}\,, \tag{23a}$$

$$\boldsymbol{P}^*(t) = e^{\boldsymbol{V}_{\boldsymbol{\theta}}(t-t_{k-1})}\boldsymbol{P}_{k-1}e^{\boldsymbol{V}'_{\boldsymbol{\theta}}(t-t_{k-1})} + \int_{t_{k-1}}^{t} e^{\boldsymbol{V}_{\boldsymbol{\theta}}(t-s)}\Delta t\boldsymbol{\beta}(\boldsymbol{m}^*(s); \boldsymbol{\theta})e^{\boldsymbol{V}'_{\boldsymbol{\theta}}(t-s)}ds\,. \tag{23b}$$

The solution for $\boldsymbol{m}^*(t)$ is obtained by applying the integrating factor method of Eq. (17) from Section S.5 to the initial value problem of Eq. (22a) using an integrating factor

$$I = e^{-\int_{t_{k-1}}^{t_k}\boldsymbol{V}_{\boldsymbol{\theta}}ds} = e^{-\boldsymbol{V}_{\boldsymbol{\theta}}(t-t_{k-1})}\,. \tag{24}$$

The solution for $\boldsymbol{P}^*(t)$ is obtained by applying the solution formula of Eq. (15), for a differential Sylvester Eq. (14), to the system described in Eq. (22b). These time-discretized solutions allow to use the update steps of a discrete-time Kalman filter (Jazwinski, 2007), whose equations are given by

2. Update: Compute the first two moments $\boldsymbol{m}_k$ and $\boldsymbol{P}_k$ of the filtering distribution at time t_k ($k = 1, \ldots, \tau$) via the following correction step

$$\begin{gathered} \boldsymbol{\mu}_k = \boldsymbol{G}_k\boldsymbol{m}^*_k\,, \quad \boldsymbol{S}_k = \boldsymbol{G}_k\boldsymbol{P}^*_k\boldsymbol{G}'_k + \boldsymbol{R}_k\,, \quad \boldsymbol{K}_k = \boldsymbol{P}^*_k\boldsymbol{G}'_k\boldsymbol{S}^{-1}_k\,, \\ \boldsymbol{m}_k = \boldsymbol{m}^*_k + \boldsymbol{K}_k(\boldsymbol{y}_k - \boldsymbol{\mu}_k)\,, \quad \boldsymbol{P}_k = \boldsymbol{P}^*_k - \boldsymbol{K}_k\boldsymbol{S}_k\boldsymbol{K}'_k\,, \end{gathered} \tag{25}$$

where $\boldsymbol{m}_k$, $\boldsymbol{P}_k$, $\boldsymbol{m}^*_k$, $\boldsymbol{P}^*_k$, $\boldsymbol{\mu}_k$ and $\boldsymbol{S}_k$ depend on $\boldsymbol{\psi}$.

3. Optimization:

For a linear Gaussian continuous-discrete state-space model the marginal likelihood of the measurements $\boldsymbol{y}_{1:\tau}$ (Mbalawata et al., 2013) is the following Gaussian distribution

$$\boldsymbol{y}_{1:\tau} \sim \mathcal{N}\left(\begin{bmatrix} \boldsymbol{\mu}_1(\boldsymbol{\psi}) \\ \vdots \\ \boldsymbol{\mu}_\tau(\boldsymbol{\psi}) \end{bmatrix}, \begin{bmatrix} \boldsymbol{S}_1(\boldsymbol{\psi}) & & \\ & \ddots & \\ & & \boldsymbol{S}_\tau(\boldsymbol{\psi}) \end{bmatrix}\right), \tag{26}$$

whose optimal parameters can be found via

$$\begin{aligned} \boldsymbol{\psi} &\leftarrow \underset{\boldsymbol{\psi}\geq\mathbf{0}}{\operatorname{argmin}} - \ell(\boldsymbol{\psi}|\boldsymbol{y}_1,\ldots,\boldsymbol{y}_\tau)\,, \\ \boldsymbol{y}_k &\sim \mathcal{N}(\boldsymbol{\mu}_k(\boldsymbol{\psi}),\boldsymbol{S}_k(\boldsymbol{\psi}))\,, \quad \forall k=1,\ldots,\tau\,, \end{aligned} \tag{27}$$

where

$$\ell(\boldsymbol{\psi}|\boldsymbol{y}_1,\ldots,\boldsymbol{y}_\tau) = -\frac{1}{2}\sum_{k=1}^{\tau}\log|2\pi\boldsymbol{S}_k| - \frac{1}{2}\sum_{k=1}^{\tau}(\boldsymbol{y}_k-\boldsymbol{\mu}_k)'\boldsymbol{S}_k^{-1}(\boldsymbol{y}_k-\boldsymbol{\mu}_k) \tag{28}$$

is the marginal loglikelihood of the measurements.

4. Smoothing: Estimate $\boldsymbol{x}_k|\boldsymbol{y}_{1:\tau} \sim \mathcal{N}(\boldsymbol{m}^s_{k|\tau},\boldsymbol{P}^s_{k|\tau})$ $(k=1,\ldots,\tau)$ using the following backward step(Jazwinski, 2007)

$$\begin{cases} \boldsymbol{B}_{k+1} = \boldsymbol{P}_k e^{\boldsymbol{V}'_{\boldsymbol{\theta}}}(\boldsymbol{P}^*_{k+1})^{-1} \\ \boldsymbol{m}^s_{k|\tau} = \boldsymbol{m}_k + \boldsymbol{B}_{k+1}(\boldsymbol{m}^s_{k+1|\tau} - \boldsymbol{m}^*_{k+1}) \\ \boldsymbol{P}^s_{k|\tau} = \boldsymbol{P}_k + \boldsymbol{B}_{k+1}(\boldsymbol{P}^s_{k+1|\tau} - \boldsymbol{P}^*_{k+1})\boldsymbol{B}'_{k+1} \end{cases} \tag{29}$$

where $e^{(\cdot)}$ is the matrix exponential operator and $\boldsymbol{m}_k$, $\boldsymbol{P}_k$, $\boldsymbol{m}^*_k$, $\boldsymbol{P}^*_k$ are those obtained from the filtering (prediction and update) steps. We use a gradient-based method to solve the optimization problem of Eq. (27). The gradient $\nabla_{\boldsymbol{\psi}}\ell(\boldsymbol{\psi}|\boldsymbol{y}_1,\ldots,\boldsymbol{y}_\tau)$ of the marginal log-likelihood of the measurements is defined by the following partial derivatives

$$\begin{aligned} -\frac{\partial\ell(\boldsymbol{\psi})}{\partial\psi_j} &= tr\left(\boldsymbol{S}^{-1}\frac{\partial\boldsymbol{S}}{\partial\psi_j}\right) - \left(\frac{\partial\boldsymbol{\mu}}{\partial\psi_j}\right)'\boldsymbol{S}^{-1}(\boldsymbol{y}-\boldsymbol{\mu})\,, \\ &-(\boldsymbol{y}-\boldsymbol{\mu})'\boldsymbol{S}^{-1}\frac{\partial\boldsymbol{S}}{\partial\psi_j}\boldsymbol{S}^{-1}(\boldsymbol{y}-\boldsymbol{\mu}) - (\boldsymbol{y}-\boldsymbol{\mu})'\boldsymbol{S}^{-1}\frac{\partial\boldsymbol{\mu}}{\partial\psi_j}\,, \end{aligned} \tag{30}$$

where

$$\boldsymbol{S} = \begin{bmatrix} \boldsymbol{S}_1 & & \\ & \ddots & \\ & & \boldsymbol{S}_\tau \end{bmatrix}, \quad \boldsymbol{y} = \begin{bmatrix} \boldsymbol{y}_1 \\ \vdots \\ \boldsymbol{y}_\tau \end{bmatrix}, \quad \boldsymbol{\mu} = \begin{bmatrix} \boldsymbol{\mu}_1 \\ \vdots \\ \boldsymbol{\mu}_\tau \end{bmatrix}. \tag{31}$$

This requires, at every time point k, $p+q$ more prediction and update steps in order to compute the terms $\frac{\partial\boldsymbol{S}_k}{\partial\theta_j}$'s, $\frac{\partial\boldsymbol{\mu}_k}{\partial\theta_j}$'s, $\frac{\partial\boldsymbol{S}_k}{\partial\rho_0}$, $\frac{\partial\boldsymbol{\mu}_k}{\partial\rho_0}$, $\frac{\partial\boldsymbol{S}_k}{\partial\rho_1}$ and $\frac{\partial\boldsymbol{\mu}_k}{\partial\rho_1}$, where p and q are the dimensions of $\boldsymbol{\theta}$ and $\boldsymbol{\rho}$. These are obtained by differentiating Eqs. (22) and (25) w.r.t. each component of $\boldsymbol{\psi}$, as shown below.

1'. Prediction derivatives step:

"$\frac{\partial\boldsymbol{m}^*}{\partial\psi_j}$": If we differentiate the system defined in Eq. (22a) w.r.t. ψ_j we get

$$\begin{cases} \frac{d}{dt}\left(\frac{\partial}{\partial\psi_j}\boldsymbol{m}^*(t)\right) = \frac{\partial}{\partial\psi_j}\boldsymbol{V}_{\boldsymbol{\theta}}\boldsymbol{m}^*(t) = \boldsymbol{V}_{\boldsymbol{\theta}}\frac{\partial}{\partial\psi_j}\boldsymbol{m}^*(t) + \left(\frac{\partial}{\partial\psi_j}\boldsymbol{V}_{\boldsymbol{\theta}}\right)\boldsymbol{m}^*(t) \\ \frac{\partial}{\partial\psi_j}\boldsymbol{m}^*(t_{k-1}) = \frac{\partial}{\partial\psi_j}\boldsymbol{m}_{k-1}\,. \end{cases} \tag{32}$$

By using the integrating factor

$$I = e^{-\int_{t_{k-1}}^{t}\boldsymbol{V}_{\boldsymbol{\theta}}ds} = e^{-\boldsymbol{V}_{\boldsymbol{\theta}}(t-t_{k-1})}\,, \tag{33}$$

we get

$$\begin{aligned}\frac{\partial \boldsymbol{m}^*}{\partial \psi_j} &= e^{\boldsymbol{V_\theta}(t-t_{k-1})}\left\{\int_{t_{k-1}}^{t} e^{-\boldsymbol{V_\theta}(s-t_{k-1})}\frac{\partial \boldsymbol{V_\theta}}{\partial \psi_j}\boldsymbol{m}^*(s)ds + \frac{\partial}{\partial \psi_j}\boldsymbol{m}_{k-1}\right\} \\ &= \int_{t_{k-1}}^{t} e^{\boldsymbol{V_\theta}(t-s)}\frac{\partial \boldsymbol{V_\theta}}{\partial \psi_j}e^{\boldsymbol{V_\theta}(s-t_{k-1})}\boldsymbol{m}_{k-1}ds + e^{\boldsymbol{V_\theta}(t-t_{k-1})}\frac{\partial}{\partial \psi_j}\boldsymbol{m}_{k-1}\,.\end{aligned} \tag{34}$$

"$\boldsymbol{\frac{\partial P^*}{\partial \psi_j}}$": By differentiating the system defined in Eq. (22b) w.r.t. ψ_j we get

$$\begin{aligned}\frac{d}{dt}\left(\frac{\partial}{\partial \psi_j}\boldsymbol{P}^*(t)\right) &= \frac{\partial}{\partial \psi_j}\left\{\boldsymbol{V_\theta P}^*(t) + \boldsymbol{P}^*(t)\boldsymbol{V}'_{\boldsymbol{\theta}} + \Delta t\boldsymbol{\beta}(\boldsymbol{m}^*(t),\boldsymbol{\theta})\right\} \\ &= \frac{\partial}{\partial \psi_j}\boldsymbol{V_\theta P}^*(t) + \boldsymbol{V_\theta}\frac{\partial}{\partial \psi_j}\boldsymbol{P}^*(t) + \frac{\partial}{\partial \psi_j}\boldsymbol{P}^*(t)\boldsymbol{V}'_{\boldsymbol{\theta}} + \boldsymbol{P}^*(t)\frac{\partial}{\partial \psi_j}\boldsymbol{V}'_{\boldsymbol{\theta}} + \\ &\quad +\Delta t\left\{\sum_{i=1}^{n}\frac{\partial \boldsymbol{\beta}(\boldsymbol{m}^*(t),\boldsymbol{\theta})}{\partial x_i}\frac{\partial m_i^*(t)}{\partial \psi_j} + \frac{\partial \boldsymbol{\beta}(\boldsymbol{m}^*(t),\boldsymbol{\theta})}{\partial \psi_j}\right\} \\ &= \boldsymbol{V_\theta}\frac{\partial}{\partial \psi_j}\boldsymbol{P}^*(t) + \frac{\partial}{\partial \psi_j}\boldsymbol{P}^*(t)\boldsymbol{V}'_{\boldsymbol{\theta}} + \boldsymbol{Q}(t),\end{aligned} \tag{35}$$

where

$$\begin{aligned}\boldsymbol{Q}(t) &= \frac{\partial}{\partial \psi_j}\boldsymbol{V_\theta P}^*(t) + \boldsymbol{P}^*(t)\frac{\partial}{\partial \psi_j}\boldsymbol{V}'_{\boldsymbol{\theta}} \\ &\quad +\Delta t\left\{\sum_{i=1}^{n}\frac{\partial \boldsymbol{\beta}(\boldsymbol{m}^*(t),\boldsymbol{\theta})}{\partial x_i}\frac{\partial m_i^*(t)}{\partial \psi_j} + \frac{\partial \boldsymbol{\beta}(\boldsymbol{m}^*(t),\boldsymbol{\theta})}{\partial \psi_j}\right\}\,,\end{aligned} \tag{36}$$

which is a differential Sylvester equation. The corresponding initial value problem is

$$\begin{cases}\frac{d}{dt}\left(\frac{\partial}{\partial \psi_j}\boldsymbol{P}^*(t)\right) = \boldsymbol{V_\theta}\frac{\partial}{\partial \psi_j}\boldsymbol{P}^*(t) + \frac{\partial}{\partial \psi_j}\boldsymbol{P}^*(t)\boldsymbol{V}'_{\boldsymbol{\theta}} + \boldsymbol{Q}(t) \\ \frac{\partial}{\partial \psi_j}\boldsymbol{P}^*(t_{k-1}) = \frac{\partial}{\partial \psi_j}\boldsymbol{P}_{k-1}\,,\end{cases} \tag{37}$$

whose solution is given, by applying Eq. (15), as

$$\begin{aligned}\frac{\partial}{\partial \psi_j}\boldsymbol{P}^*(t) &= e^{(t-t_{k-1})\boldsymbol{V_\theta}}\frac{\partial}{\partial \psi_j}\boldsymbol{P}_{k-1}e^{(t-t_{k-1})\boldsymbol{V}'_{\boldsymbol{\theta}}} \\ &\quad + \int_{t_{k-1}}^{t} e^{(t-s)\boldsymbol{V_\theta}}\boldsymbol{Q}(s)e^{(t-s)\boldsymbol{V}'_{\boldsymbol{\theta}}}ds\,.\end{aligned} \tag{38}$$

2'. Update derivatives step:
The resulting solutions $\frac{\partial \boldsymbol{m}_k^*}{\partial \psi_j}$ and $\frac{\partial \boldsymbol{P}_k^*}{\partial \psi_j}$, $(k = 1, \ldots, \tau)$ are then used to update the corresponding initial values via a set of equations obtained by differentiating Eq. (25) w.r.t. each component of $\boldsymbol{\psi}$, that is

$$\begin{aligned}&\frac{\partial \boldsymbol{\mu}_k}{\partial \psi_j} = \boldsymbol{G}_k\frac{\partial \boldsymbol{m}_k^*}{\partial \psi_j}\,, \quad \frac{\partial \boldsymbol{S}_k}{\partial \psi_j} = \boldsymbol{G}_k\frac{\partial \boldsymbol{P}_k^*}{\partial \psi_j}\boldsymbol{G}'_k + \frac{\partial \boldsymbol{R}_k}{\partial \psi_j}\,, \\ &\frac{\partial \boldsymbol{K}_k}{\partial \psi_j} = \frac{\partial \boldsymbol{P}_k^*}{\partial \psi_j}\boldsymbol{G}'_k\boldsymbol{S}_k^{-1} - \boldsymbol{P}_k^*\boldsymbol{G}'_k\boldsymbol{S}_k^{-1}\frac{\partial \boldsymbol{S}_k}{\partial \psi_j}\boldsymbol{S}_k^{-1}\,, \\ &\frac{\partial \boldsymbol{m}_k}{\partial \psi_j} = \frac{\partial \boldsymbol{m}_k^*}{\partial \psi_j} + \frac{\partial \boldsymbol{K}_k}{\partial \psi_j}(\boldsymbol{y}_k - \boldsymbol{\mu}_k) - \boldsymbol{K}_k\frac{\partial \boldsymbol{\mu}_k}{\partial \psi_j}\,, \\ &\frac{\partial \boldsymbol{P}_k}{\partial \psi_j} = \frac{\partial \boldsymbol{P}_k^*}{\partial \psi_j} - \frac{\partial \boldsymbol{K}_k}{\partial \psi_j}\boldsymbol{S}_k\boldsymbol{K}'_k - \boldsymbol{K}_k\frac{\partial \boldsymbol{S}_k}{\partial \psi_j}\boldsymbol{K}'_k - \boldsymbol{K}_k\boldsymbol{S}_k\frac{\partial \boldsymbol{K}_k}{\partial \psi_j}'\,.\end{aligned} \tag{39}$$

Input: $\{\boldsymbol{x}_k\}_k$, $\boldsymbol{V}$, $\boldsymbol{h}(\boldsymbol{x},\boldsymbol{\theta})$, $\boldsymbol{x}_0 \sim \mathcal{N}_n(\boldsymbol{m}_0, \boldsymbol{P}_0)$
Output: $\hat{\boldsymbol{\psi}}_{ekf}$, $\boldsymbol{m}^s_{k|\tau}$ and $\boldsymbol{P}^s_{k|\tau}$, $(k = 1, \ldots, \tau)$
while $\epsilon > tol$ **do**
  $\boldsymbol{\psi}_{old} \leftarrow \boldsymbol{\psi}$
  for $k = 1 : \tau$ **do**
    1. Prediction: get $\boldsymbol{m}^*_k$ and $\boldsymbol{P}^*_k$
    1'. Prediction derivatives: get $\frac{\partial \boldsymbol{m}^*_k}{\partial \psi_j}$, $\frac{\partial}{\partial \psi_j}\boldsymbol{P}^*_k$
    2. Update: get $\boldsymbol{m}_k$, $\boldsymbol{P}_k$, $\boldsymbol{\mu}_k$ and $\boldsymbol{S}_k$
    2'. Update derivatives: get $\frac{\partial \boldsymbol{m}_k}{\partial \psi_j}$, $\frac{\partial}{\partial \psi_j}\boldsymbol{P}_k$, $\frac{\partial \boldsymbol{\mu}_k}{\partial \psi_j}$, $\frac{\partial}{\partial \psi_j}\boldsymbol{S}_k$
  end
  3. Optimization: $\boldsymbol{\psi} \leftarrow \underset{\boldsymbol{\psi}>0}{\text{argmin}} - \ell\left(\boldsymbol{\psi}|\boldsymbol{y}_{1:\tau}\right)$
  4. Smoothing: Get $\boldsymbol{m}^s_{k|\tau}$ and $\boldsymbol{P}^s_{k|\tau}$, $(k = 1, \ldots, \tau)$
  5. Update prior for $\boldsymbol{x}_0$: $\boldsymbol{m}_0 \leftarrow \boldsymbol{m}^s_{1|\tau}$ and $\boldsymbol{P}_0 \leftarrow \boldsymbol{P}^s_{1|\tau}$
  $\epsilon \leftarrow \frac{\|\boldsymbol{\psi}-\boldsymbol{\psi}_{old}\|_2}{\|\boldsymbol{\psi}_{old}\|_2}$
end

Algorithm S.2: Pseudocode of the inference procedure.

$\lambda_{HSC\to P1}$	$\lambda_{HSC\to P2}$	$\lambda_{P1\to A}$	$\lambda_{P1\to B}$	$\lambda_{P1\to C}$	$\lambda_{P2\to D}$	$\lambda_{P2\to E}$	
6.60	3.48	2.2	2.24	2.16	1.8	1.68	
δ_A	δ_B	δ_C	δ_D	δ_E	α_{HSC}	δ_{P1}	δ_{P2}
2.52	2.232	2.304	1.224	1.512	6.24	3.4912	3.2728

Table S.1: Values of the dynamic vector parameter $\boldsymbol{\theta}$ used in the simulations of Section "**Validation and comparison with the prior art**" from the main paper.

All the results obtained from every prediction/update step at time point t_k $(k = 1, \ldots, \tau)$, along with the corresponding derivatives, are then used to compute the marginal log-likelihood of the measurements $\ell(\boldsymbol{\psi}|\boldsymbol{y}_1, \ldots, \boldsymbol{y}_\tau)$ and its gradient which, in turn, are used for the optimization step of Eq. (27). The proposed inference procedure is summarised in Algorithm S.2. All the integrals involved for the computation of $\boldsymbol{P}^*_k$, $\frac{\partial}{\partial \psi_j}\boldsymbol{m}^*_k$, $\frac{\partial}{\partial \psi_j}\boldsymbol{P}^*_k$ are estimated numerically using a 3rd-order Gauss-Legendre method (Davis and Rabinowitz, 2007).

S.7 Validation and comparison with the prior art

We report here additional results on the simulation studies from Section "**Validation and comparison with the prior art**" from the main paper. The values of the dynamic vector parameter $\boldsymbol{\theta}$ used for the simulations are reported in Table S.1. Each clonal trajectory has been simulated from $t_0 = 0$ to $t = 1$ with a time-step increment Δt, from which we selected a subset of time points with a sampling frequency τ. Information on the different combinations of number of independent simulations n_{sim}, the number of clones n_{clones}, the time increment Δt, the sampling frequency τ, the fraction of false-negatives ζ, the measurement noise parameters ρ_0, ρ_1, and the initial condition for the state vector $\boldsymbol{x}_0$ used for the simulations are reported in Table S.2. Results from Fig. S.1 clearly indicate that our proposed method overall outperforms the other candidates. In particular, Figs. S.1a-c suggest that our proposed method is the most robust against

n_{sim}	n_{clones}	Δt	τ	ζ	ρ_0	ρ_1	$\boldsymbol{x}_0$
100	3	0.001	4	0%	0.1	0.1	100 for HSC, 0 otherwise
100	3	0.001	7	0%	0.1	0.1	100 for HSC, 0 otherwise
100	3	0.001	15	0%	0.1	0.1	100 for HSC, 0 otherwise
100	3	0.001	7	10%	0.1	0.1	100 for HSC, 0 otherwise
100	3	0.001	7	50%	0.1	0.1	100 for HSC, 0 otherwise
100	3	0.001	7	90%	0.1	0.1	100 for HSC, 0 otherwise
100	3	0.001	7	0%	0	0	100 for HSC, 0 otherwise
100	3	0.001	7	0%	1	1	100 for HSC, 0 otherwise
100	3	0.001	7	0%	10	10	100 for HSC, 0 otherwise

Table S.2: Different combinations of values for the number of independent simulations n_{sim}, the number of clones n_{clones}, the time increment Δt, the sampling frequency τ, the fraction of false-negatives ζ, the measurement noise parameters ρ_0, ρ_1, and the initial condition for the state vector $\boldsymbol{x}_0$ used in the simulations of Section "**Validation and comparison with the prior art**" from the main paper.

false negative errors compared to the other methods, which provide more biased estimates for the dynamic parameters $\boldsymbol{\theta}$, as we increased the fraction ζ of missing data. Plot panels d-f show that decreasing the sampling frequency τ of the simulated trajectories did not affect the estimates provided by our proposed method, whereas those obtained with any of the competitor approaches were increasingly biased. Finally, after increasing the measurement noise through the parameters ρ_0 and ρ_1, our proposed method still provided better estimates compared to the other candidate methods.

S.8 Model misspecification

We report here the details on the parameters used for the simulations of Section "**Model misspecification**" from the main paper. The values of the dynamic vector parameter $\boldsymbol{\theta}$ of both candidate models used for the simulations are reported in Table S.3. Each clonal trajectory has been simulated from $t_0 = 0$ to $t = 1$ with a time-step increment Δt, from which we selected a subset of time points with a sampling frequency τ. Information on the number of independent simulations n_{sim}, the number of clones n_{clones}, the time increment Δt, the sampling frequency τ, the fraction of false-negatives ζ, the measurement noise parameters ρ_0, ρ_1, and the initial condition for the state vector $\boldsymbol{x}_0$ can be found in Table S.4.

S.9 Scalability to complex networks

We report here the details on the parameters used for the simulations of Section "**Scalability to complex networks**" from the main paper. The values of the dynamic vector parameter $\boldsymbol{\theta}$ used for the simulation are reported in Table S.5. Each clonal trajectory has been simulated from $t_0 = 0$ to $t = 1$ with a time-step increment Δt, from which we selected a subset of time points with a sampling frequency τ. Information on the number of independent simulations n_{sim}, the number of clones n_{clones}, the time increment Δt, the sampling frequency τ, the fraction of false-negatives ζ, the measurement noise parameters ρ_0, ρ_1, and the initial condition for the state vector $\boldsymbol{x}_0$ can be found in Table S.6.

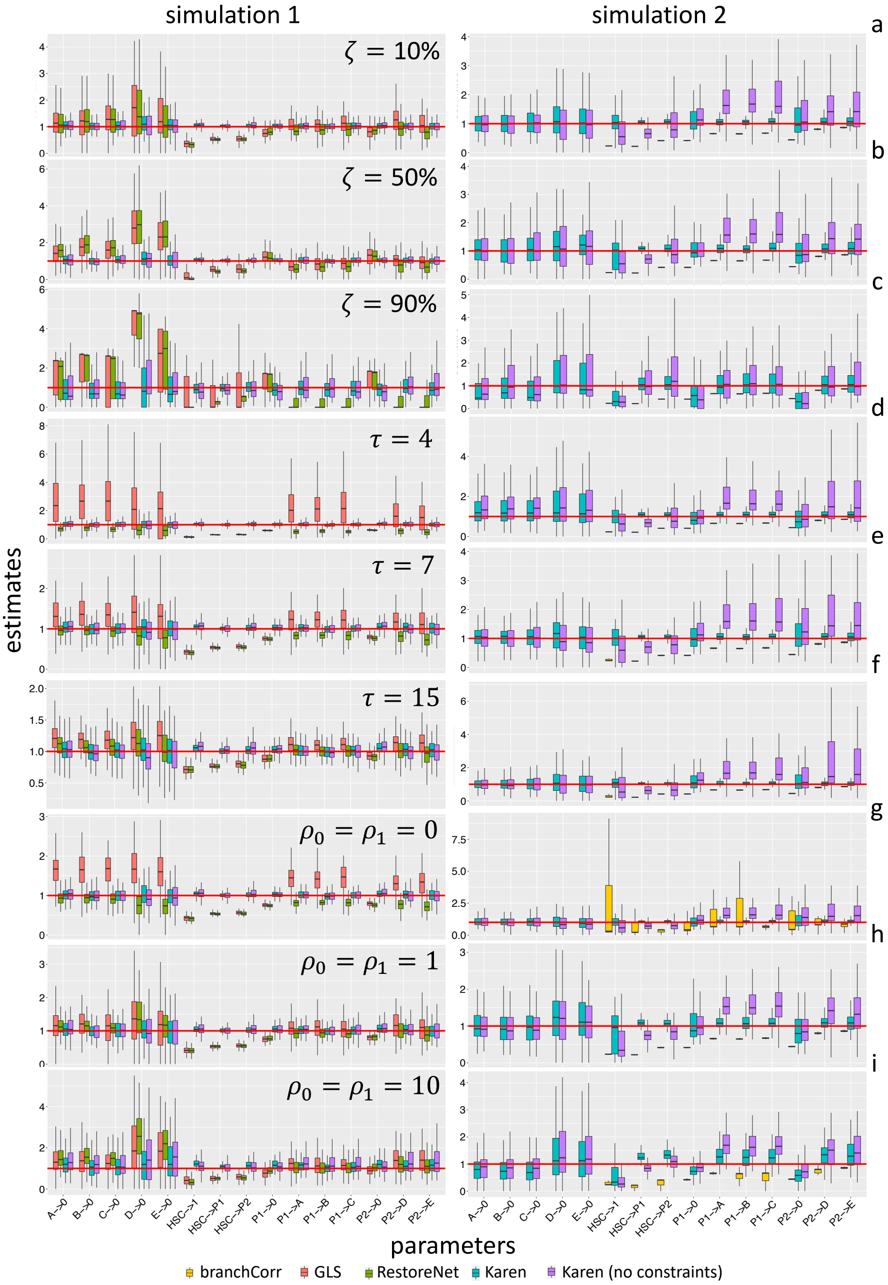

Figure S.1: For each synthetic study with observed (left) and unobserved (right) progenitor cells HSC, P1 and P2: the boxplots (y-axis) of the estimated parameters divided by the true parameters for each reaction rate (x-axis) obtained from each method (colors), across all simulations, under different values of the fraction ζ of unobserved data, the sampling frequency τ, and the noise parameters ρ_0 and ρ_1.

model 1						
$\lambda_{P1\to T}$	$\lambda_{P1\to B}$	$\lambda_{P1\to NK}$	$\lambda_{P2\to G}$	$\lambda_{P2\to M}$	δ_T	δ_B
0.65	0.9	0.925	0.975	0.55	3.5	3.1
δ_{NK}	δ_G	δ_M	α_{HSC}	α_{P1}	α_{P2}	
4	3.7	4.1	0.25	0.225	0.275	

model 2							
$\lambda_{P1\to T}$	$\lambda_{P1\to B}$	$\lambda_{P2\to NK}$	$\lambda_{P2\to G}$	$\lambda_{P2\to M}$	$\lambda_{P3\to B}$	$\lambda_{P3\to NK}$	δ_T
0.65	0.9	0.925	0.975	0.55	0.6	0.7	3.5
δ_B	δ_{NK}	δ_G	δ_M	α_{HSC}	α_{P1}	α_{P2}	α_{P3}
3.1	4	3.7	4.1	0.25	0.225	0.275	0.24

Table S.3: Values of the dynamic vector parameter $\boldsymbol{\theta}$ of both candidate models used in the simulations of Section "**Model misspecification**" from the main paper.

n_{sim}	n_{clones}	Δt	τ	ζ	ρ_0	ρ_1	$\boldsymbol{x}_0$
100	3	0.001	15	0%	0.1	0.5	100 for HSC, 0 otherwise

Table S.4: Values of the number of independent simulations n_{sim}, the number of clones n_{clones}, the time increment Δt, the sampling frequency τ, the fraction of false-negatives ζ, the measurement noise parameters ρ_0, ρ_1, and the initial condition for the state vector $\boldsymbol{x}_0$ used in the simulations of Section "**Model misspecification**" from the main paper.

$\lambda_{MEP\to P}$	$\lambda_{MEP\to ERY}$	$\lambda_{GMP\to G}$	$\lambda_{GMP\to M}$	$\lambda_{CLP\to NK}$
1.62	1.344	1.35	1.53	1.68
$\lambda_{CLP\to B}$	$\lambda_{CLP\to T}$	α_{HSC}	α_{MPP}	α_{CMP}
2.24	2.44	1.82	1.62	1.26
α_{CLP}	α_{MEP}	α_{GMP}	δ_P	δ_{ERY}
1.46	1.42	0.64	2.775	2.67
δ_G	δ_M	δ_T	δ_B	δ_{NK}
2.81	2.91	11.5	2.54	4.91

Table S.5: Values of the dynamic vector parameter $\boldsymbol{\theta}$ used in the simulations of Section "**Scalability to complex networks**" from the main paper.

n_{sim}	n_{clones}	Δt	τ	ζ	ρ_0	ρ_1	$\boldsymbol{x}_0$
50	100	0.001	5	80%	0.1	0.1	100 for HSC, 0 otherwise

Table S.6: Values of the number of independent simulations n_{sim}, the number of clones n_{clones}, the time increment Δt, the sampling frequency τ, the fraction of false-negatives ζ, the measurement noise parameters ρ_0, ρ_1, and the initial condition for the state vector $\boldsymbol{x}_0$ used in the simulations of Section "**Scalability to complex networks**" from the main paper.

S.10 Computational complexity

We evaluated the computational complexity of our proposed inference framework Karen in several synthetic studies. For the simulations we used the cell differentiation structure from Fig. 2b of the main manuscript as the true data generative process. We implemented the linear constraints of Eq. (16), and we varied the fraction of unobserved data (ζ), the sampling frequency (τ), the

$\lambda_{P1\to T}$	$\lambda_{P1\to B}$	$\lambda_{P1\to NK}$	$\lambda_{P2\to G}$	$\lambda_{P2\to M}$	δ_T	δ_B
0.65	0.9	0.925	0.975	0.55	3.5	3.1
δ_{NK}	δ_G	δ_M	α_{HSC}	α_{P1}	α_{P2}	
4	3.7	4.1	0.25	0.225	0.275	

Table S.7: Values of the dynamic vector parameter $\boldsymbol{\theta}$ used in the simulation study of computational complexity.

Δt	ρ_0	ρ_1	$\boldsymbol{x}_0$
0.001	0.1	0.5	100 for HSC, 0 otherwise

Table S.8: Values for the time increment Δt, the measurement noise parameters ρ_0, ρ_1, and the initial condition for the state vector $\boldsymbol{x}_0$ used in the simulation study of computational complexity.

ζ	τ	n_{Cl}	threads	RAM (Mb)	max used (Mb)	run time (sec.)
10%	7	100	1	42.2	116.2	3515.09
50%	7	100	1	42.2	116.2	3083.50
90%	7	100	1	42.2	116.6	2956.12
70%	7	10	1	3.3	17.4	559.90
70%	7	100	1	42.1	116.7	2542.97
70%	7	1000	1	3910.7	13673.7	22999.16
70%	7	1000	2	1958.5	5622.1	14640.42
70%	7	1000	4	982	2832.9	7080.80
70%	7	1000	10	396.10	1012.20	2990.80
70%	7	1000	16	249.80	562.30	2397.40
70%	7	1000	20	200.70	375.30	1869.11
70%	5	10	1	3.20	19.80	413.70
70%	10	10	1	3.50	17.20	721.23
70%	20	10	1	3.90	22.90	1569.02
70%	30	10	1	4.40	18.80	1878.61

Table S.9: Memory usage (RAM) in megabytes (Mb), the maximum memory used (max used) since last call to `gc(reset = TRUE)`, and the run time (in seconds) by varying the fraction of unobserved data (ζ), the sampling frequency (τ), the number of clones (n_{Cl}), and the number of CPUs (threads).

number of clones (n_{Cl}), and the number of CPUs (threads). The values of the dynamic vector parameter $\boldsymbol{\theta}$ used for the simulations are reported in Table S.7. Each clonal trajectory has been simulated from $t_0 = 0$ to $t = 1$ with a time-step increment Δt, from which we selected a subset of time points with a sampling frequency τ. Information on the time increment Δt, the measurement noise parameters ρ_0, ρ_1, and the initial condition for the state vector $\boldsymbol{x}_0$ can be found in Table S.8. The run times (in seconds) and the RAM memory usage (in megabytes) resulting from the simulation study are reported in Table S.9.

S.11 Genotoxicity data rescaling

In this section we report the details on the methods used to rescale the data analysed in Section "**Genotoxicity study**" from the main paper. Clonal track-

	PGK				SFV			
	DNA	VCN	PS	SD	DNA	VCN	PS	SD
Min.	8.64	1.31	1	60	8.64	0.240	1	189
1st Qu.	106.56	10.90	2	1969	94.50	5.320	1	1130
Median	200.00	13.59	2	5881	200.00	6.300	2	2973
Mean 1	181.07	12.80	1.96	9351	222.88	6.219	2.1	4695
3rd Qu.	200.25	13.90	2	14055	222.50	7.800	3	7390
Max.	973.00	27.00	3	49853	973.00	10.500	7	15375

Table S.10: Mice study: Quartiles and range of the confounders (inner columns) separately for PGK and SFV treatments (outer columns).

ing samples were collected under heterogeneous technical conditions as reported in Table S.10, thus making data not directly comparable across time and cell types. Here we consider the DNA amount (in nanograms), the vector copy number (VCN), the pool size (PS) and the PCR protocol as potential confounders. By analogy to the shape-constrained splines (SCS) method (Del Core et al., 2022), we first evaluate and then remove the effect of the confounders from the observed data using a regression approach. More precisely, we first perform a log-link Poisson regression on the collected cell counts $\boldsymbol{y}$ against the corresponding confounding factors and the possibly factors of interest, leading to the following model

$$log(\boldsymbol{\lambda}) = \boldsymbol{X}\boldsymbol{\beta}\,, \quad y_i \sim \text{Poisson}(\lambda_i)\,, \tag{40}$$

where y_i is the i-th component of $\boldsymbol{y}$, λ_i is the i-th component of $\boldsymbol{\lambda}$ for $i = 1, \ldots, n$, $\boldsymbol{X} = \begin{bmatrix}\mathbf{1} & \boldsymbol{X}_c\end{bmatrix}$ is the full design matrix including a term $\mathbf{1} \in \mathbb{R}^{n\times 1}$ for the intercept and a term $\boldsymbol{X}_c \in \mathbb{R}^{n\times 4}$ with confounder-specific columns. Parameter estimates $\hat{\boldsymbol{\beta}} = \left(\hat{\beta}_0, \hat{\boldsymbol{\beta}}_c'\right)'$ are obtained with a Fisher scoring algorithm, and the rescaled clonal tracking data has been defined as the partial residuals corresponding to the confounders, that is

$$\boldsymbol{y}^{res} = \exp\left(\log(\boldsymbol{y}) - \boldsymbol{X}_c\hat{\boldsymbol{\beta}}_c\right)\,, \tag{41}$$

where $\hat{\boldsymbol{\beta}}_c$ are the optimal parameters for the confounders.

S.12 Rhesus macaque data rescaling

In this section we report the details on the methods used to rescale the data analysed in Section "**Rhesus Macaques study**" from the main paper. Although the sample DNA amount was maintained constant during the whole experiment (200 ng for ZH33 and ZG66 or 500 ng for ZH17), the sample collected resulted in different magnitudes of total number of reads. Table S.11 shows the total number of reads collected in each sample of the rhesus macaque clonal tracking dataset. This discrepancy makes all the samples not directly comparable across time and cell types. Therefore we define the rescaled barcode counts y_{ijk}^{res} as

$$y_{ijk}^{res} = y_{ijk}w_{ij}\,, \quad w_{ij} = \frac{\min_{lm}\sum_n y_{lmn}}{\sum_n y_{ijn}}\,, \tag{42}$$

	time (months)	T	B	NK	M	G
ZH33	1	1465289	74735	135092	119331	2831
	2	225797	216844	335789	1035270	908685
	3	243986	413757	663184	886682	816990
	4.5	485542	479493	834064	985821	987171
	6.5	645005	676413	926089	895309	911637
	9.5	829073	962325	1057398	1229233	1220506
ZH17	1	51802	1347050	1288718	1351450	707382
	2	826190	1342700	1350703	1354355	1213749
	3	1303922	1347692	1338024	1347177	1283250
	4.5	190591	1206361	489098	572877	1195585
	6.5	887851	610999	1344488	381552	1339299
ZG66	1	752127	0	211350	13382	0
	2	692133	58890	308800	363310	145252
	3	339292	209137	424458	808404	704331
	4.5	617281	338977	718472	887183	897672

Table S.11: Rhesus macaque study: total number of reads (sum across the clones) collected from each animal (outer rows) at each time point (inner rows) and for all the cell types (columns).

where y_{ijk} is the ijk-entry of the barcode matrix with dimensions (i, j, k) mapping respectively time, cell type and clone. The weights w_{ij}s ensure that all the clones of cell type j collected at time i are directly comparable with those collected at different time points and for different cell types, even though the corresponding samples have different total number of reads.

References

Adams, R., et al. *Calculus: A Complete Course.* Pearson Canada., 2021.

Behr, M., et al. Solution formulas for differential sylvester and lyapunov equations. *Calcolo*, 56(4):51, 2019. doi: 10.1007/s10092-019-0348-x.

Davis, P. J. and Rabinowitz, P. *Methods of numerical integration.* Courier Corporation, 2007.

Del Core, L., et al. Normalization of clonal diversity in gene therapy studies using shape constrained splines. *Scientific Reports*, 12(1):3836, 2022. doi: 10.1038/s41598-022-05837-0.

Érdi, P. and Tóth, J. *Mathematical models of chemical reactions: theory and applications of deterministic and stochastic models.* Manchester University Press, 1989.

Jazwinski, A. H. *Stochastic processes and filtering theory.* Courier Corporation, 2007.

Kloeden, P. and Platen, E. *Numerical Solution of Stochastic Differential Equations.* Stochastic Modelling and Applied Probability. Springer Berlin Heidelberg, 2011.

Mbalawata, I. S., et al. Parameter estimation in stochastic differential equations with markov chain monte carlo and non-linear kalman filtering. *Computational Statistics*, 28(3):1195–1223, 2013. doi: 10.1007/s00180-012-0352-y.

Sjöberg, P., et al. Fokker–planck approximation of the master equation in molecular biology. *Computing and Visualization in Science*, 12(1):37–50, 2009. doi: 10.1007/s00791-006-0045-6.
